# Development of a holistic urban heat island evaluation methodology

**DOI:** 10.1038/s41598-020-75018-4

**Published:** 2020-10-21

**Authors:** Valentino Sangiorgio, Francesco Fiorito, Mattheos Santamouris

**Affiliations:** 1 DICATECH, Politecnico di Bari, Via Edoardo Orabona 4, Bari, Italy; 2 High Performance Architecture, School of Built Environment, University of New South Wales, Sydney, NSW 2052 Australia

**Keywords:** Atmospheric science, Climate change, Environmental impact, Natural hazards

## Abstract

Urban Heat Island (UHI) phenomenon concerns the development of higher ambient temperatures in urban districts compared to the surrounding rural areas. Several studies investigated the influence of individual parameters in the UHI phenomenon, on the other hand, an exhaustive study that quantifies the influence of each parameter in the resulting UHI is missing in the related literature. This paper proposes a new index aimed at quantifying the hazard of the absolute maximum UHI intensity in urban districts during the Summer season by taking all the parameters influencing the phenomenon into account. In addition, for the first time, the influence of each parameter has been quantified. City albedo and the presence of greenery represent the most important characteristics with an influence of 29% and 21%. Population density, width of streets, canyon orientation and building height has a medium influence of 12%, 10%, 9% and 8% respectively. The remaining parameters have an overall influence of 11%. These results are achieved by exploiting three synergistically related techniques: the Analytic Hierarchy Processes to analyse the parameters involved in the UHI phenomenon; a state-of-the-art technique to acquire a large set of data; and an optimization procedure involving a involving a Jackknife resampling approach to calibrate the index by exploiting the effective UHI intensity measured in a total of 41 urban districts and 35 European Cities.

## Introduction

Global warming and the associated rise in extreme temperatures substantially increase the possibility of heat waves jeopardising the safety of some vulnerable population classes. In this context, the Urban Heat Island (UHI) phenomenon aggravates warm temperatures in urban districts together with the related risks^1^. To this aim, the scientific and technical communities are interested in studying the phenomenon in depth, in order to obtain new procedures to quantify the potential Urban Heat Island Intensity (UHII) and predict the hazard. The hazard of the phenomenon is connected to many *classes of parameters* including: (1) *meteorological variables* such as synoptic weather and climate conditions, (2) *characteristics of the city*, intended as urban layout and materials' characteristics, (3) *anthropogenic heat*, related to population density (4) and *city canyons* whose influence depend on urban layout. In addition, the measured UHII depends on the selection of the reference rural measuring station and in particular on the characteristics of the urban districts where the station is located^2^.

Several authors demonstrated that UHI has a serious impact on safety of some vulnerable population classes, and buildings energy consumption principally during the Summer period^3^. Indeed, the phenomenon highly increases the cooling energy consumption and the corresponding peak electricity demand of the cities^4^. Consequently, the UHI can be associated with an important increase of urban pollutants concentration, it is related with the tropospheric ozone^5^ and the city's carbon footprint^6^. Finally, the phenomenon seriously affects comfort, health and increases mortality problems^7–9^.

In the building sector, scientist and technicians are interested in developing new techniques and procedures to evaluate the average UHII, and maximum UHII in order to forecast and mitigate this effect, decrease energy demand of building stock and eradicate the energy poverty^10^. This objective is framed within the European and global challenge of innovation for the built environment to assumes a minimization of the energy consumption of buildings and mitigate of the urban heat island and the local climate change.

On the other hand, the classical evaluation of the absolute maximum UHII can be complex and resource consuming. In addition, data acquisition strategy varies considerably among the reported studies making complex an interstudy comparison. In particular, the necessary data acquisition presents critical points: (1) high duration of the experimental phase; (2) high number of measuring stations used; (3) data acquirement depending on the seasons; (4) difficulty in finding historical data to improve the efficacy of monitoring phase in order to observe and support climatological studies of local climate variations^4,11^. In this context, an index calibrated to forecast the absolute maximum UHII phenomenon would be of great support for technicians of city government and for civil protection in order to analyse the territory and define mitigation strategies.

In the related literature there are some attempts to obtain a concise equation to associate some parameters related to the urban districts with the increase of the heat island effect. The first attempt regards the dependencies of UHII with the immediate environment of the measurement site such as the ratio between building height and width of street (H/W)^12^. Successively, more in dept investigation focused on the sky view factor (SVF) as discussed in the review study of Unger^13^. The ratio H/W and the SVF sky view factor have the advantages to be very easy to be used by practitioners. On the other hand, H/W and SVF efficiency is local and does not include other important factors. Indeed, in recent studies, the challenge is integrating other types of variables such as common surface types in cities or greenery. Because of the complex processes governing the urban climate, involving qualitative and quantitative data, the related literature exhibits few attempts to find an equation to estimate the potential UHI. In addition, typically such approaches must be calibrated for each new city and for this reason they have very limited applicability^14–16^. An effective attempt to involve multi parameters has been developed by Theeuwes et al.^17^. Unfortunately, the authors themselves specified that their approach is not able to consider important urban properties, such as building materials or anthropogenic heat. To sum up, a calibration of an index that manages to consider several parameters including *meteorological variables, characteristics of the city*, *anthropogenic heat*, and *city canyons* would be extremely useful in the scientific and technical community, but it has not yet been obtained in previous researches.

Among the existing methodologies to calibrate an intensity index, the Multi-Criteria Decision-Making (MCDM) approaches are particularly effective to consider both qualitative and quantitative data in the analysis^18^. In particular, the Analytic Hierarchy Processes (AHP) has been widely used in the literature to understand hazard and risk phenomena as specified in the review work of De Almeida et al.^19^. In addition, optimization procedures can be applied to calibrate the index and support the MCDM approaches^20,21^. To this aim, such methodology can be suitable in calibrating an index of potential absolute maximum UHII, but this approach has not yet been attempted in the related literature.

In this paper, the aims of the current study are twofold: for the first time an exhaustive analysis is performed to quantify the influence of parameters affecting the UHII during the Summer season. In addition, the work proposes the calibration of a novel index involving many parameters devoted to quantifying the potential absolute maximum Urban Heat Island Intensity (*I*_*UHII*_) in urban districts in order to predict the local hazard of the phenomenon.

This ambitious objective is achieved by employing four synergistically related techniques: (1) the AHP to analyse, structure the problem and define the index; (2) a large data acquisition process to obtain an exhaustive dataset; (3) an optimization procedure to calibrate the index based on a large data acquisition of 41 urban districts; (4) two validation test involving a Jackknife approach to identify the stability of the solution and the *Absolute Error* of the proposed model.

## Results

### Influence of parameters affecting absolute max UHII

For the first time, we achieved the quantification of the influence of every parameter for the creation of the UHI phenomenon in urban districts. In particular, the weights of eleven parameters or criteria *i* (with *i* = 1,…,11) are defined and calibrated by using an AHP and Optimization-based calibration.

The resulting influence is expressed in percentage and the description of results follows the parameters classification proposed in the introduction.

The most important parameters belong to the *characteristics of the city* class and in particular to the Land Cover Types: (1) “Albedo” (*i* = 5) represents the ability of urban districts surfaces to reflect solar radiation and has an influence of 29%; (2) the “Greenery” (*i* = 6) percentage presents an influence of 21%.

Another important class regards the presence of *city canyons* in urban districts. Indeed, the average “width of street” (*i* = 9), “building height” (*i* = 8) and the “canyon orientation” (*i* = 10) affect the phenomenon of 10%, 8% and 9% respectively, with a further increase of 5% for the “irregularity of the city” streets (*i* = 11).

The *anthropogenic heat* is a class that affects the phenomenon with an intermediate level. The “population density” (*i* = 7) is the only associated parameter of this class with 12% of influence.

Finally, the *meteorological variables* have the least influence of all the parameters: (1) “Clear sky days” (*i* = 4) with a value of 2%, (2) “Windless days” (*i* = 1) with 2%, (3) “Average max Summer temperature” (*i* = 2) and “average Summer thermal excursion” (*i* = 3) of 1% each one.

**Figure 1:**
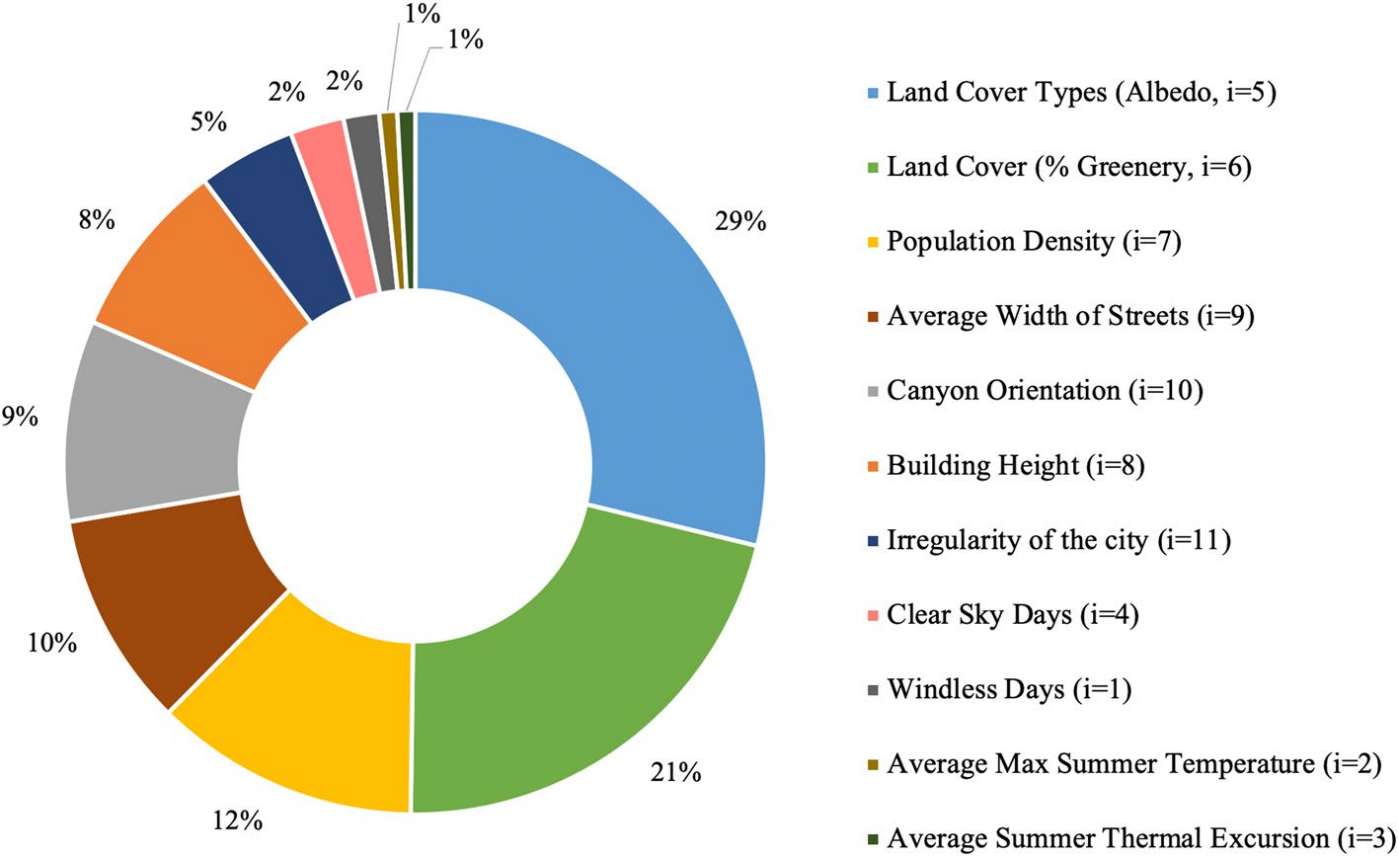
Iinfluence of each parameter in the absolute max UHII phenomenon.

Figure 1 shows the influence of every parameters expressed in percentage and displayed in a pie chart.

### The novel index aimed at quantifying potential absolute max UHII in urban districts

We also developed a novel index *I*_*UHII*_ to predict the potential UHII in urban districts based on the same eleven parameters, hereafter named criteria in accordance with AHP^22^. In addition, for each *criterion i* a set of intensity ranges *j* (with *j* = 1,…,*n*_*i*_) is defined to characterize its intensity levels.

Our results show that *I*_*UHII*_ is easy to be applied by practitioners thanks to a simple and effective equation:

1 $${\varvec{I}}_{{{\varvec{UHII}}}} = v_{1} {*}w_{1j} + v_{2} *w_{2j} + v_{3} *w_{3j} + \ldots + v_{11} {*}w_{11j}$$

were *v*_*i*_ and *w*_*ij*_ are the weights associated to the criteria *i* and to the intensity ranges *j* respectively.

Suitable tabulated weights (Supplementary Table S1) are obtained to use the Eq. (1) where every single parameter (or criterion) is associated to a weight *v*_*i*_ and every intensity ranges is associated to a specific weight *w*_*ij*_.

These tabulated weights are calibrated by exploiting a set of 41 urban districts ($Ud=1,\dots ,41$) for which the real max UHII is obtained from an exhaustive bibliographic analysis. A total of 35 European cities have been involved to contemplate different climate zone, different cities typologies in order to get the calibrated weights effective in different contexts.

### Validation results

**Figure 2:**
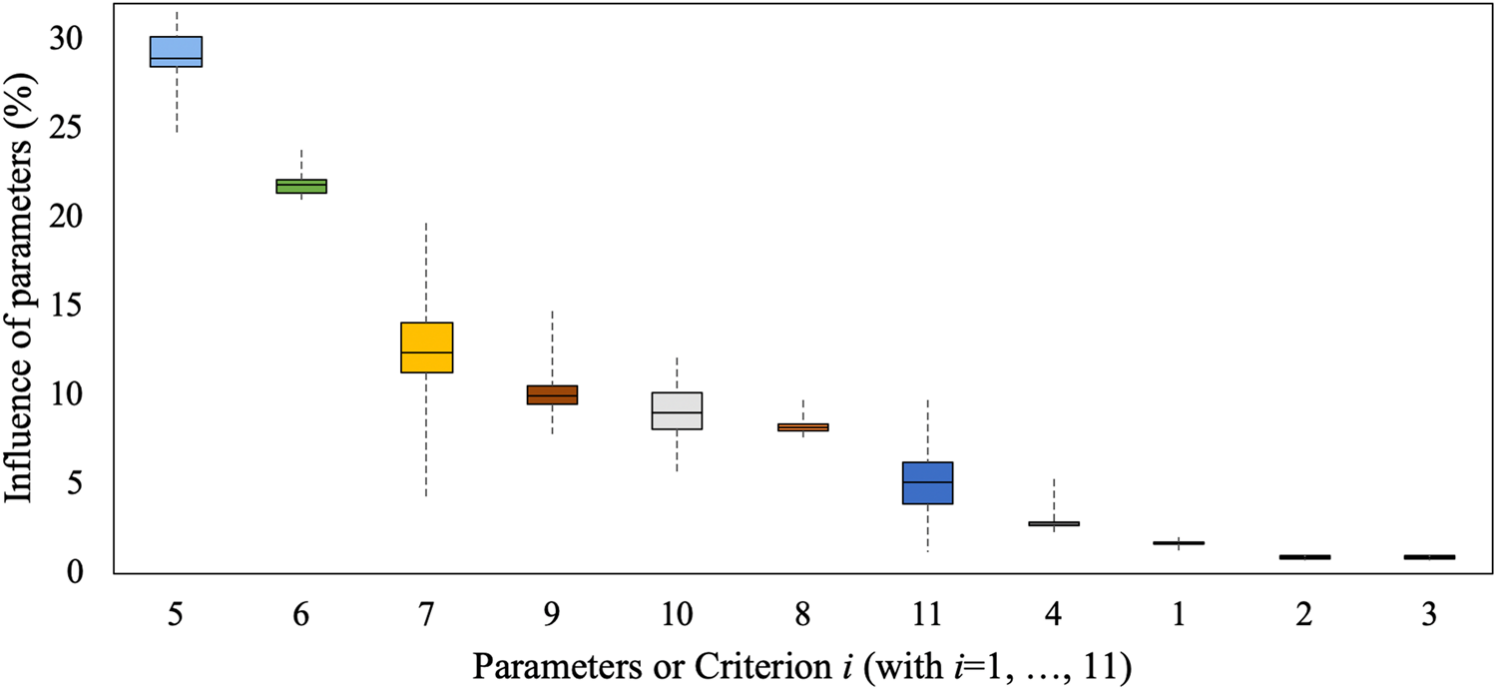
Boxplot of the influence of each parameter (or criterion) in the absolute max UHII phenomenon.

To validate the index, a comparison of the prediction (obtained with the index ${{\varvec{I}}}_{{\varvec{U}}{\varvec{H}}{\varvec{I}}{\varvec{I}}}$) with all the 41 independent values is carried out. In addition, a Jackknife approach (a one-by-one removal of the 41 urban districts) is used in order to evaluate the stability of the solution. This validation shows that the calibration is robust and, even if the input data changes due to the one-by-one removal, the results do not vary significantly. Moreover, Fig. 2 shows the statistical graph (Boxplot) of each parameter influence in the absolute max UHII phenomenon. In particular, the coloured boxes represent the distribution of the weights (expressed in percentage) and the black horizontal line inside the boxes denotes the median of the sample. While the box contains all the results within the 25th and 75th percentile of the population, the vertical dotted line contains all the results which are not considered outliers.

**Figure 3:**
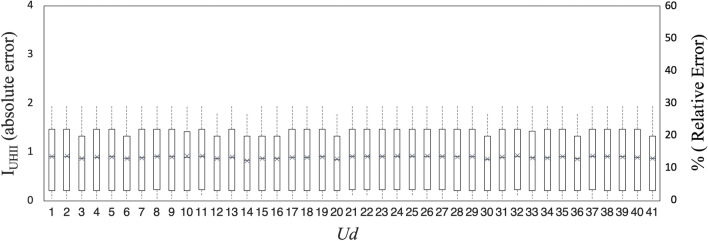
Analysis of the *Absolute Error* and *Relative Error* (average values represented by the symbol “ × ”) for each urban district removal with the Jackknife approach.

In addition, in order to explicitly prove the effectiveness of the proposed calibration by a common metrics, a second analysis identifies the *Absolute Error* and *Relative Error* of ${{\varvec{I}}}_{{\varvec{U}}{\varvec{H}}{\varvec{I}}{\varvec{I}}}$ in comparison with the effective absolute max UHII obtained from the bibliography. Also in this case, the Jackknife approach is used to perform a resampling of the input data and verify the robustness of the solution. Figure 3 shows the values of the absolute error and the mean relative error (represented in the same graph by the symbol “ × ”) obtained for every removal of the 41 urban districts. It is worth noting that the average values of both absolute and relative errors do not change significantly despite the resampling of input data, proving the robustness of the obtained results.

This additional analysis demonstrates that the proposed model is able to identify the potential absolute max UHII with an average accuracy of about 1 °C (*Mean Absolute Error* = 0.9 °C) and an average mean relative error less than fifteen percent (*Mean Relative Error* = 14.5%). In particular, in more than the 70% of the investigated urban district the *Relative Error* is less than 10%. On the other and, in the Urban district where the absolute max UHII is small, often there is a higher error (above 15%) even if the *Absolute Error* remain about 0.9 °C.

These obtained error can be considered acceptable to the effective application of the index. Indeed, it is in line with the reliability that characterizes the UHII measurements with standard fixed station. Furthermore, the classical approach to measure UHII can be affected by an inaccuracy due to the choice of the rural station measurement (to be compared with the city station measurement), as it is discussed by Oke et al.^2^.

A third analysis, based on the *Pearson correlation coefficient (ρ),* is used to verify the importance of all the considered parameters involved in the proposed AHP framework and consequently in the index *I*_*UHII*_. In particular, the Optimization-based calibration showed that the *meteorological variables* have the minor influence of all the parameters on the development of the absolute max UHII. On the other and, the four considered meteorological drivers are appropriately correlated and then are important to describe the phenomenon as emphasized by the *Pearson correlation coefficient* (*Windless days ρ* = *0.308*, *Average max Summer temperature ρ* = *0.272* and *Average Summer thermal excursion ρ* = *0.238* and *Clear sky days ρ* = *0.240)*.

This third analysis provides the scatter plot of every parameter (criterion *i*) in relation with the absolute max UHII, together with the corresponding correlation coefficients. Supplementary Fig. S1 shows the complete overview of the relationships between variables and the investigated phenomenon. This analysis confirms that most of the involved parameters have a medium or high correlation and none of the involved parameters has very low and a null correlation. Hence, the related criteria can be considered important in the absolute max UHII manifestation.

In addition, in order to show how the proposed method assess the absolute max UHII in different cities, an additional scatter plot with observed values versus predicted values is showed in Supplementary Fig. S2. The *Pearson coefficient* (*ρ* = *0.716*) exhibits a good correlation providing a further information about the effectiveness of the calibration.

## Discussion

The present study provides a comprehensive analysis of all the parameters involved in the absolute max UHI phenomenon, an effective data collection procedure and a useful Index, calibrated and validated, to forecast the absolute max UHII in urban districts. This discussion focuses on the principal two contribution of the proposed work (the analysis of the involved parameters and the new index) in comparison with previous studies. Afterwards, the limitations are discussed, and the application potential of the proposed method are emphasized by showing the case of Milan (Italy).

The first contribution of this work regards an exhaustive analysis of the parameters involved in the absolute max UHII by classifying them in criteria, intensity ranges and variables. In the related literature these parameters have been often studied individually or by considering all the parameters associated with a specific “*class”*: *meteorological variables*, *characteristics of the city*, *anthropogenic heat*, and *city canyons*.

To provide some examples, Zhao et al.^23^ and Lokoshchenko^24^ investigate the contributions of local *meteorological variables* in the UHI. In particular, Zhao et al.^23^ focus on the influence of humid and dry climates with specific investigation on the precipitation effect. However, Lokoshchenko^24^ investigates UHII changes over the past hundred years principally studying the correlation of the phenomenon with the urban dry island and including a connection with the growth of population density.

The *characteristics of the city* are principally investigated in studies involving the use of remote sensing to evaluate the UHII^25,26^. These studies confirm the importance of vegetation and albedo connected with the built environment and only marginally consider other classes of parameters including average air temperature and anthropogenic heat fluxes.

On the contrary in other researches*, anthropogenic heat* is a topic of great interest, and many studies are completely focused on the development of new approaches for the evaluation of heat flux database, with high spatial resolution^27–29^.

In addition, more local analysis are developed to study *city canyons.* To provide an example an exhaustive review about the sky view factor in *city canyons* is discussed in Unger^13^.

Such literature framework suggests that studies of the UHI are principally focused on the investigation of few parameters. More in details, some existing studies that consider a greater number of parameters are typically dedicated on the investigation of specific regions or cities and do not propose a quantification of their influence. For instance, Lokoshchenko^30^ investigates many meteorological parameters such as: air temperature, soil temperature, precipitation, wind speed, but also green areas, albedo and density of population. However, Lokoshchenko^30^ focusses the attention on the UHI in Moscow and do not quantify the impact of the considered parameters in the phenomenon. In this context the review papers of Santamouris^4,10^, demonstrate that a complete analysis on the parameters influencing the absolute maximum UHII is missing in related literature. To this reason, in comparison with these previous studies, the proposed work provides an important new contribution by quantifying the influence of the parameters involved in the phenomenon on the basis of a scientific approach never applied before in this field. Indeed, this research contemplates the use of multi-criteria methods in synergy with mathematical optimization and a Jackknife resampling approach to investigate the absolute maximum UHII.

The second topic of the proposed work regards the achieving of a new index to evaluate the hazard of the absolute max UHI phenomenon in urban district during the Summer season. A similar goal is proposed in the study of Theeuwes et al.^17^. Such study calibrates a diagnostic equation of the max UHI by using many parameters including meteorological variables, such as incoming shortwave radiation, daily thermal excursion and wind speed. The Theeuwes’s study utilized weather stations set-up by hobby meteorologists located in gardens. However, such approach gives a limited variability in urban climate zones where measurements are taken. In addition, the obtained diagnostic equation does not consider several other urban properties such as building materials, anthropogenic heat and characteristics of city and canyons. Alternatively, other studies applied statistical models and linear regression for the calibration including parameters related to the characteristics of city in addition to the meteorological variables^15,16^. Unfortunately, these last statistical approaches require a retuning for each city as it is remarked in the study of Theeuwes et al.^17^.

To sum up, in the existing approaches, an easy-to-use concise index, able to consider many parameters influencing the phenomenon, and applicable in different cities, has never been obtained. On the contrary, our research deals with the calibration and validation of an index involving both qualitative and quantitative data in the analysis. In addition, in order to overcome the drawback of retuning, the proposed index is calibrated by exploiting a comparison with the effective absolute max UHII recorded by standard measuring stations in 41 urban districts of 32 different cities located in the European continent. The effective annual max UHII is obtained by a wide literature analysis considering only data obtained by standard measuring stations and published in peer review journals, books, and research reports. In particular, the weights of criteria of the proposed index are calibrated by solving an optimization problem to minimize the differences between the results of the *Index of Potential UHII* (*I*_*UHII*_) and the effective annual max UHII. The mathematical optimization problem exploits the results of the AHP and the dataset to calibrate the index and quantify the influence of every parameter. Finally, two validation tests are performed involving a Jackknife approach to identify the stability of the solution and the evaluation of the *Absolute Error* of the proposed model.

The shortcoming and limitation of the proposed procedure regards the applicability for the winter season or in arctic climate. Some existing researches studied the mechanisms of the UHI in this context, showing some differences with the Summer UHI, such as the relevant influence of the soil temperature and the improving of the hazard for low temperatures. In addition, also the effect of UHI on society is different, indeed in arctic climate, the positive effects are proven due to the reduction of the need for heating^31,32^. However, currently the related literature does not provide enough data to perform an exhaustive calibration even in this cold weather context. For this reason, the current study is limited on the evaluation of the absolute max UHII in Summer. Moreover, the presented study does not consider some parameters typically influencing the UHI in cold climate.

On the other hand, the proposed empirical approach has many advantages in comparison with the approaches mentioned above. It is easy to apply for practitioners and the necessary data for application are available without complex acquisition procedures or unopened access databases. In particular, a comprehensive dataset to forecast the max UHII should contain at least the following information: (1) meteorological variables that can be extracted from existing meteorological European database^33^ and Copernicus data^34^; (3) characteristics of the city acquirable by exploiting satellite data processing and image analysis; (4) anthropogenic heat and city canyons that can be extracted from European Environment Agency^35^, Google Maps and Technical cartographies (when available).

The resulting index is able to outcome a hazard value to forecast the absolute max UHII in the urban districts by using eleven input data including meteorological variables, characteristics of the city (in terms of albedo and greenery), anthropogenic heat and city canyons.

To provide an example, there are not exhaustive studies on the heat island effect for the whole city Milan in in related literature. If a practitioner wanted to know the potential Hazard of all the districts of Milan, the proposed index can be applied as showed as follows.

The collection procedure allows to easily achieve the input data of all the 88 urban districts of the city of Milan. Note that information are acquired by considering an area within 500 m from the centre of gravity of considered districts.

For the case of Milan, data regarding *meteorological variables* are the same for the whole city, instead the *characteristics of the city*, *anthropogenic heat* and *city canyons* have a great variability.

**Figure 4:**
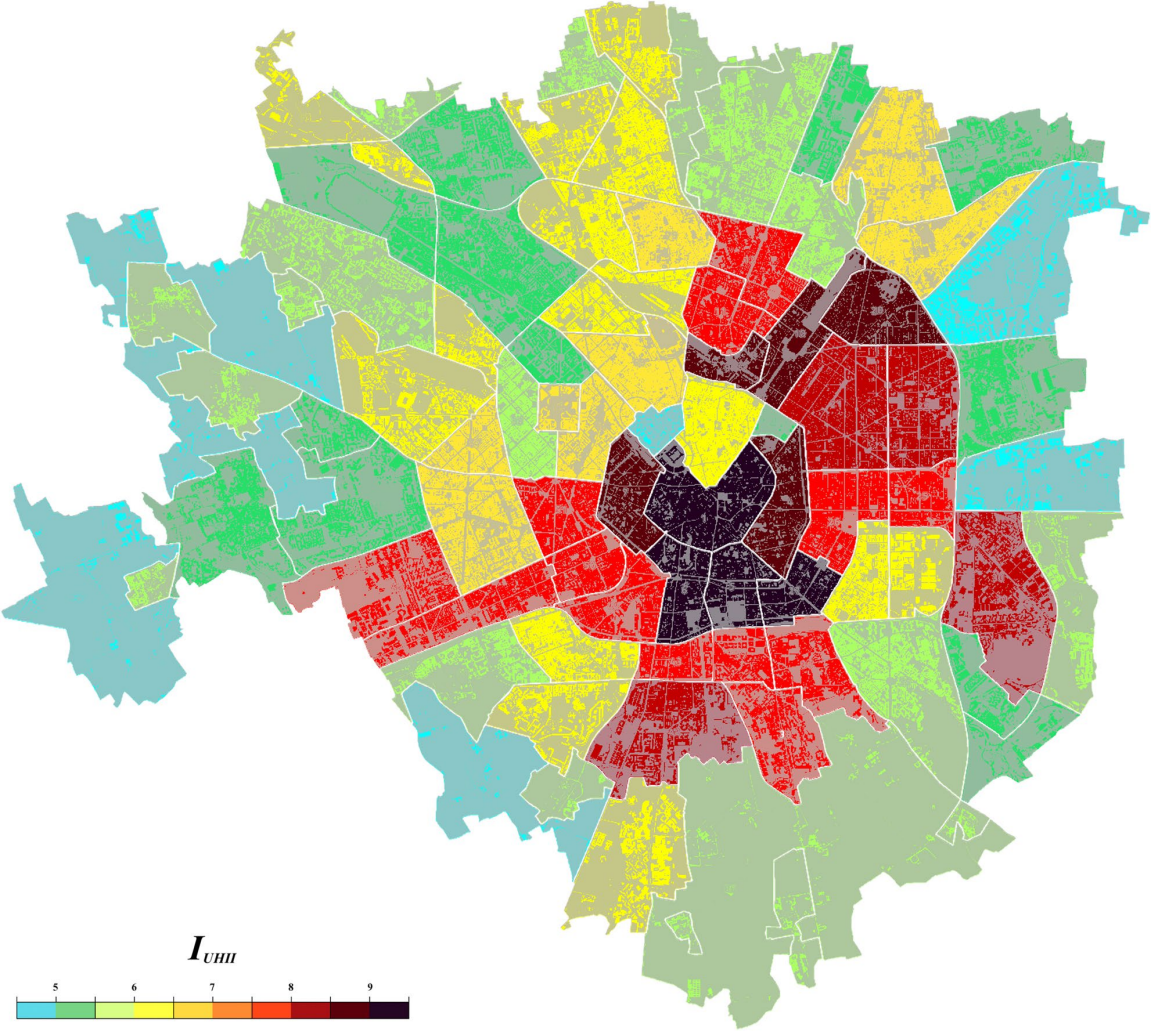
Example of the application of the *I*_*UHII*_ to create a hazard map of the city districts of Milan. The Map is generated by using SketchUp Make 2016 v16.1.1451 (https://www.sketchup.com/) and Adobe Photoshop 2020 v21.1.0 (https://www.adobe.com) starting from the imagery of the “Territorial information system” of Milano (https://geoportale.comune.milano.it/sit/).

The proposed index *I*_*UHII*_ is applied to forecast the hazard of the phenomenon for every one of the 88 urban districts by using Eq. (1) and the tabulated weight of Supplementary Table S1. Figure 4 shows the application of the index to generate an hazard map of the city of Milan for the Absolute Max UHII. The central areas of Milan, (near the cathedral) are the most subject to suffer the UHI caused by an intensely developed urban fabric. The only two exceptions concern the central districts with large parks (Sempione Park and the Garden of Porta Venezia). On the contrary, outside the central area, the UHI effect decreases principally thanks to greater green areas and a minor canyon effect produced by different urban layout (e.g. lower buildings, wider and greener streets). To sum up, the proposed approach can be useful to analyse the UHI phenomenon in single urban district or in hole cities. It is a useful tool for researcher to forecast the UHI hazard during in Summer season. In addition, also practitioners, administrations and governments can exploit this tool for urban planning, to understand the needs and effectively design new green areas or mitigation strategies of UHI as showed in the case of Milano.

## Conclusions

The obtained potential UHII index can be a useful tool for evaluate effective urban planning strategies at the urban scale and identify the best intervention for individual building aimed at the minimization of the phenomenon. Beyond this, the proposed index could also be used by researchers, architects, engineers and interested stakeholders to obtain maps of Hazard at different scales (urban, regional or national) and have valuable assistance in identifying energy retrofit interventions in order to improve the energy performances of buildings and mitigate the UHI.

In comparison with the existing studies, the main novelty of the paper is threefold. For the first time, the influence of each parameter involved in the UHII is quantified. Different techniques of multi-criteria decision methods, state of the art data acquisition procedure, mathematical optimization and validation are simultaneously employed in a structured and synergic way for analysing the UHII. The second novel issue regards the proposal of a quantitative index to evaluate the potential absolute max UHII. This index overcomes the limitations of the necessary experimental data acquisition described in literature to obtain a preliminary local analysis of the UHII phenomenon^4,11^.

Thirdly, the research reaches the ambitious result of calibrating the index by analysing 41 urban districts in 35 different cities, including the effective UHII registered and derived from a wide literature analysis.

The project involved a combination of interdisciplinary skills including meteorology, knowledge of UHII phenomenon, statistics, optimization and multi-criteria analysis, data mining, satellite data processing and image analysis in order to apply three synergistic techniques (AHP, a large-scale data acquisition process, an optimization and validation procedure).

Future research will evaluate a vulnerability index and an exposure index to the UHII phenomenon in order to obtain an overall risk index of the phenomenon. In addition, the proposed index will be integrated in Spatial Decision Support Systems for the large-scale risk assessment useful to set effective mitigation strategies.

## Methods

### AHP to analyse the problem

The AHP methodology is used to define the UHII index. By exploiting the information extracted from the related literature, it is possible to define criteria and sub-criteria and determine the index (An exhaustive description of the AHP method applied to define the index is presented in the supplementary methodology).

**Figure 5:**
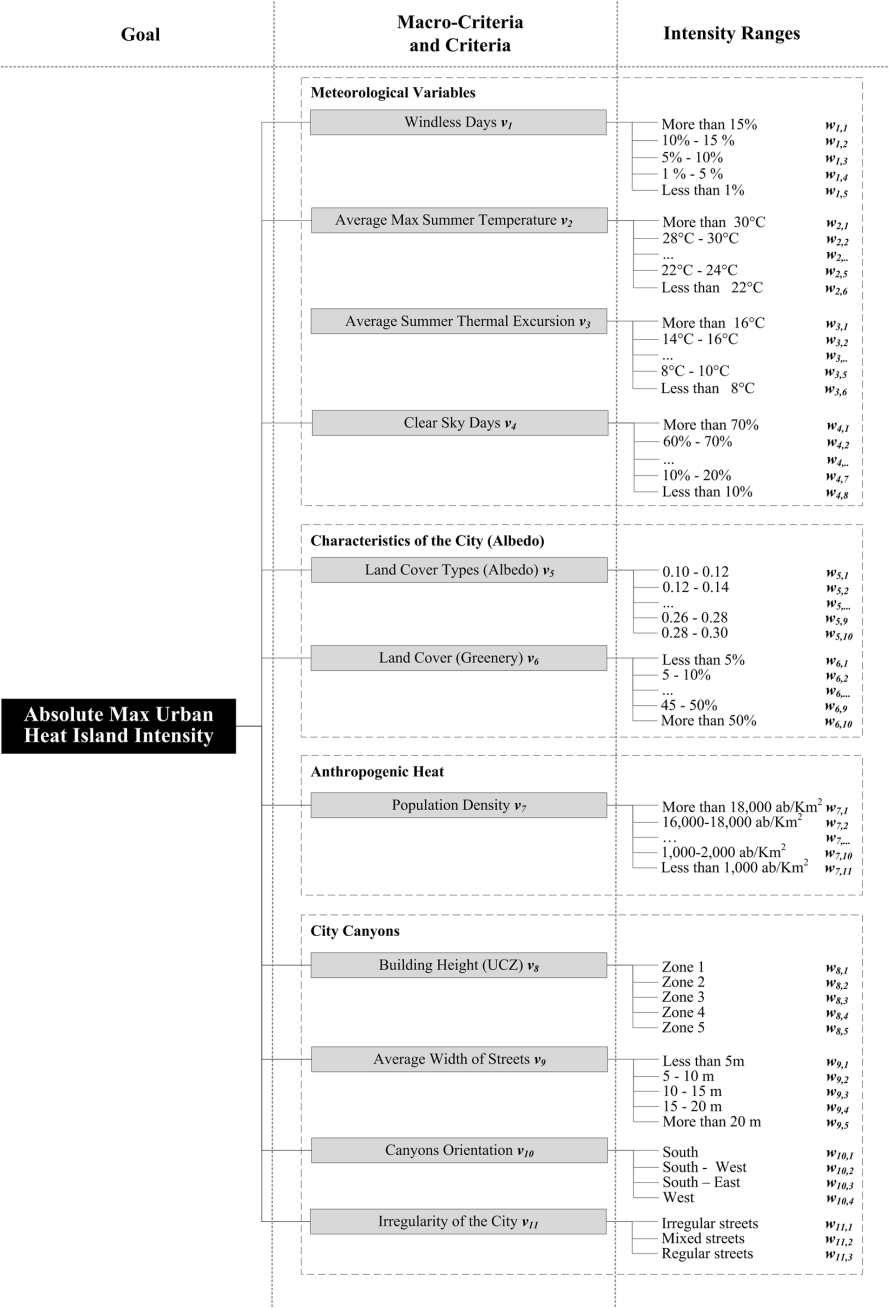
*Structure of the Problem*: weights, criteria and intensity ranges to determine an index able to quantify the potential absolute max Urban Heat Island Intensity in urban district. The chart is drawn by using Microsoft Visio 2007 v 12.0.4581.1014 (https://www.microsoft.com).

The AHP step 1 in consists in the *Structure of the Problem* to determine an index useful to quantify potential UHII in urban district. In particular, the goal is defined as the phenomenon *Absolute Max Urban Heat Island Intensity*. To this aim, eleven *criteria i* (with *i* = 1,…,11) involved in the generation of the UHI^36^ are defined and grouped into four *macro-criteria* in accordance with the classes of parameters defined in the introduction. For each *criterion* a set of intensity ranges *j* (with *j* = 1,…,*n*_*i*_) is defined to characterize its intensity levels. The eleven *criteria*, and related *intensity ranges* involved in the UHI phenomena are structured in a hierarchical flowchart that is showed in Fig. 5 and discussed in the supplementary methodology.

The AHP step 2 is used to individually analyses each aspect of the defined UHII problem (Fig. 5) in order to weight the parameters involved. The *criteria* and *intensity ranges* weights are defined as follows:

- *v*_*i*_ is the weight associated with each *i*th *criterion*
- *w*_*ij*_ is the weight associated with each *j*th *intensity range* related to the *i*th criterion.

eleven *judgment matrices A* are evaluated in order to identify the tabulated weights of *intensity ranges w*_*ij*_.

After weighting, the potential *Absolute Max* Urban Heat Island Intensity Index (*I*_*me*_) can be defined. This operation coincides with the AHP step 3 of the summary of priority. The Eq. (1) is obtained by multiplying each *criteria* weight by the *intensity range* weight and adding the results, as in the classical AHP procedure.

### The data acquisition

This section explains the data acquisition process. State of the art techniques including bibliographic research, data mining for data extraction from existing databases, satellite data processing and image analysis are applied in order to achieve an effective dataset, useful for calibrating and validating the index. In addition, modern and updated databases are used, and out random cross-checks are carried out to verify the data reliability. It is essential to specify that data regarding meteorological, city, urban district and anthropogenic parameters refers to the specific temporal period of the acquisition of the max UHII registered in the associated urban districts. The complete set of data, including the rural station geographic coordinates and the acquisition period, is showed in the Supplementary Table S2. In particular, the data acquisition process can be summarized in the identification of: the *effective UHII*, *meteorological variables*, *characteristics of the city*, *anthropogenic heat*, and *city canyons.* The acquired data regards different climatic context, dimension and characteristics of the city to achieve an effective calibration and validation.

**Figure 6:**
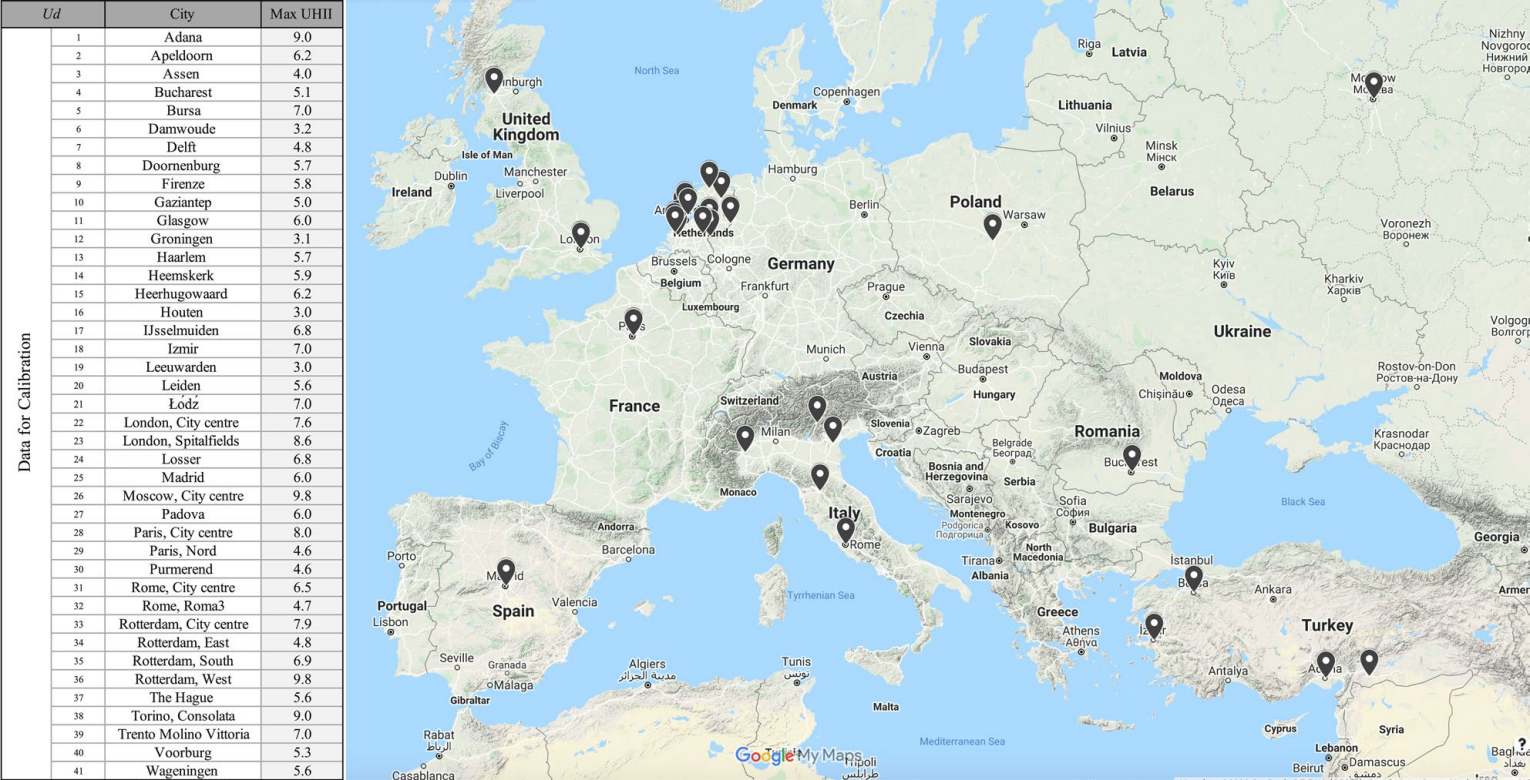
UHIIs extracted from a literature review. The figure was created by using Google My Maps 2020 (https://www.google.com/maps/d/.), Microsoft Excel v16.33 (https://www.microsoft.com) and Adobe Photoshop 2020 v21.1.0 (https://www.adobe.com).

- Experimental data on the *effective UHII* is acquired for 41 different cities in 10 different countries of the European Continent. The information is collected from 15 scientific articles published in peer review journals, books, and research reports (Table 1). Among more than hundreds investigated studies, only articles providing sufficient information on the spatial area considered, the type of experiments and the used equipment are taken into account. Many of the considered studies reported experiments and data for more than one district, thus in total the paper includes data from 41 observations.
  In addition, only studies aiming to quantify absolute maximum UHII by using fixed standard measuring equipment/station are considered while articles investigating UHI characteristics using mobile surveys techniques are rejected. To sum up, the considered studies can be considered reliable and homogeneous if they provide: (1) the absolute maximum UHII; (2) UHII acquisition performed by standard fixed measurement stations; (3) geographical coordinates of the urban station; (4) geographical coordinates of the rural stations; (5) data acquired in a period of time in which also climate data are available in the used meteorological databases.
  Moreover, Fig. 6 shows the data about specific urban districts, the related geographic position and the UHII measures. For every urban district a specific identification code $Ud=1,\dots ,41$ is assigned.
- The *meteorological variables* are extracted from the European database of Tutiempo Network^33^ and Clara^50^. This database is comprehensive since it has stored over the years many historical meteorological data of the main cities of the European continent. In addition, in order to verify the data reliability, a cross check with the database of Urbansis and Copernicus^34^ and weatherspark^51^ is exploited.
- The *characteristics of the cities* are acquired by exploiting satellite data processing and image analysis. The goal of this data acquisition technique is twofold: (1) identifying the average of the land cover albedo on the basis of the surface material typology including water, greenery, red brick roof, white plaster or pigment, gravel or concrete; (2) quantifying the percentage of greenery in relation to the evapotranspiration. In particular, an area within 500 m from the standard measuring station is considered and high-resolution imagery of Landsat 7 and Landsat 8 satellite are used.
  Supplementary Fig. S3 schematizes a part of the image analysis process for the urban district *Ud* = 32 (Rome, Roma 3, Italy). In particular, land cover type (Albedo) of the urban district is obtained by using the typical urban materials albedo initially investigated by Oke^12^ and reported in Van Hove et al.^38^ and Bradley et al.^52^. To provide an example, the most common values for albedo detected in the investigated urban districts are: green areas and grass (0.205), forests (0.150) asphalt (0.125), water (0.500), white plaster (0.500) and red brick (0.300). The results of the analysis are showed in the Supplementary Table S2.
- The *anthropogenic heat* is represented by the single criterion “population density”. Data regarding the population density can be obtained by exploiting the Eurostat^53^ and integrating some missing data of small Netherland cities by a specific search on local web-sites.
- The last information to be acquired regards the *city canyons*: these data are both quantitative and qualitative. The quantitative data regard the average width of streets and building height. The width of streets can be obtained by technical cartography when available, or from a suitable image analysis. Indeed, once extracted the asphalt cover from the area within 500 m from the measuring stations, it is possible identify the average width of streets by averaging the measurable amplitudes. The building height is obtained by exploiting the classification of Oke^54^ including the five Urban Climate Zones (UCZ) associated to the number of floors of the considered buildings. A qualitative evaluation is carried out by identifying the UCZ through Google street view or the 3D view of Google Earth when available. In addition, some qualitative analyses can be performed to evaluate the canyon orientation, by considering the directions of the main streets of the urban district, and the irregularity of the city.

**Table 1:** Scientific articles selected among the literature to extract data of absolute max UHII.

*Ud*	Country	City	Max UHII	Bibliographic Analysis
1	Turkey	Adana	9	Tayan and Toros^47^
2	Nederland	Apeldoorn	6.2	Van Hove et al.^38^
3	Nederland	Assen	4	Van Hove et al.^38^
4	Romania	Bucharest	5.1	Cheval et al.^48^
5	Turkey	Bursa	7	Tayan and Toros^47^
6	Nederland	Damwoude	3.2	Van Hove et al.^38^
7	Nederland	Delft	4.8	Van Hove et al.^38^
8	Nederland	Doornenburg	5.7	Van Hove et al.^38^
9	Italy	Firenze	5.8	Petralli et al.^44^
10	Turkey	Gaziantep	5	Tayan and Toros^47^
11	Scotland	Glasgow	6	Krüger and Emmanuel^46^
12	Nederland	Groningen	3.1	Van Hove et al.^38^
13	Nederland	Haarlem	5.7	Van Hove et al.^38^
14	Nederland	Heemskerk	5.9	Van Hove et al.^38^
15	Nederland	Heerhugowaard	6.2	Van Hove et al.^38^
16	Nederland	Houten	3	Van Hove et al.^38^
17	Nederland	IJsselmuiden	6.8	Van Hove et al.^38^
18	Turkey	Izmir	7	Tayan and Toros^47^
19	Nederland	Leeuwarden	3	Van Hove et al.^38^
20	Nederland	Leiden	5.6	Van Hove et al.^38^
21	Poland	Łódź	7	Fortuniak et al.^31^
22	UK	London, City centre	7.6	Kolokotroni and Giridharan^45^
23	UK	London, Spitalfields	8.6	Kolokotroni and Giridharan^45^
24	Nederland	Losser	6.8	Van Hove et al.^38^
25	Spain	Madrid	6	Santamouris^10^
26	Russia	Moscow, City centre	9.8	Varentsov et al.^37^
27	Italy	Padova	6	Busato et al.^43^
28	France	Paris, City centre	8	Lemonsu and Masson^49^
29	France	Paris, Nord	4.6	Lemonsu and Masson^49^
30	Nederland	Purmerend	4.6	Van Hove et al.^38^
31	Italy	Rome, City centre	6.5	Marando et al.^41^
32	Italy	Rome, Roma3	4.7	Guattari et al.^40^
33	Nederland	Rotterdam, City centre	7.9	Van Hove et al.^38^
34	Nederland	Rotterdam, East	4.8	Van Hove et al.^38^
35	Nederland	Rotterdam, South	6.9	Van Hove et al.^38^
36	Nederland	Rotterdam, West	9.8	Van Hove et al.^38^
37	Nederland	The Hague	5.6	Van Hove et al.^38^
38	Italy	Torino, Consolata	9	Milelli^42^
39	Italy	Trento Molino Vittoria	7	Giovannini et al.^39^
40	Nederland	Voorburg	5.3	Van Hove et al.^38^
41	Nederland	Wageningen	5.6	Van Hove et al.^38^

## Calibration and validation

In this section, the dataset obtained from the previous phases is used to achieve the calibration and validation of the index. Starting from the definition of the index in Eq. (1), a mathematical programming problem is formulated in order to obtain calibrated weights by an optimization procedure by exploiting 41 urban districts. Afterwards two validation tests involving a Jackknife resampling procedure are carried out to identify the stability of the solution and evaluate the *Absolute Error* and the *Mean Relative Error* of the proposed model. In addition, *Pearson correlation coefficient* of the involved *criteria* with the absolute maximum UHII is calculated, in order to verify that all the eleven *criteria* are sufficiently correlated and then important for the analysis.

In this section, the results obtained from the previous phases are used to achieve the calibration of the index.

In order to obtain the weights *v*_*i*_and calibrate the index of UHII, defined in Eq. (1), the $K$=41 urban districts ($Ud=1,\dots ,K$) are considered in eight cities of the European continent. The index of ${{I}_{UHII}}^{Ud}$ associated with the urban districts $Ud=1,\dots ,K$ is written in function of the column vector of weights ***v*** = [*v*_*1*_*, v*_*2*_*, v*_*3*_*, v*_*4*_*, v*_*5,*_* v*_*6,*_* v*_*7,*_* v*_*8,*_* v*_*9,*_* v*_*10,*_* v*_*11*_]^T^ as follows:

2 $$I_{UHII}^{Ud} \left( {\varvec{v}} \right) = v_{1} {*}w_{1j} + v_{2} *w_{2j} + v_{3} *w_{3j} + \ldots + v_{11} {*}w_{11j}$$

where *j* are the alternatives associated with the *Ud*^*-th*^ urban districts and weights *w*_*ij*_ are identified by exploiting the data showed in Supplementary Table S2 and the tabulated weight of Table S1.

In addition, the effective UHII (of every urban districts $Ud$) useful for calibration is hereafter expressed with ${UHII}^{Ud}$ ($Ud=1,\dots ,K$).

Subsequently, it is possible to define the function *F*(***v***), which evaluates the difference between the values of ${{I}_{UHII}}^{Ud}$ and the effective max ${UHII}^{Ud}$ (reported in Fig. 5) calculated for each examined urban districts *Ud* = 1,…,*K*:

3 $$F\left( {\varvec{v}} \right) = \mathop \sum \limits_{Ud = 1}^{K} \left( {I_{UHII}^{Ud} \left( {\varvec{v}} \right) - UHII^{Ud} } \right)^{2}$$

To calibrate vector ***v,*** it is assumed that the index of potential ${{I}_{UHII}}^{Ud}$ should be as close as possible to the index of effective max Urban Heat Island Intensity ${UHII}^{Ud}$ for the considered urban districts.

*F*(***v***) represents the sum of the differences between the proposed index and the effective intensity of the registered phenomenon. Consequently, *F*(***v***) is set as the objective function to be minimized by satisfying a set of constraints on vector ***v***:

4 $${\Gamma }({\varvec{v}}): \; v_{i} \ge 0\;{\text{for}}\;i = {1}, \ldots ,{11}$$

Constraints (5) are set to avoid negative or null values of the weights. Now, in order to calculate vector ***v***, the following Mathematical Programming (MP) problem is formulated:

5 $$\min \;F({\varvec{v}})$$

6 $${\text{subject}}\;{\text{to}} \;{\Gamma }({\varvec{v}})$$

A generalized reduced gradient method^55^ is applied to solve the MP problem by using a Multi-start of a population of 1000 and a convergence of 0.001.

To validate the index the following two tests and one correlation analysis are carried out:

Firstly, a specific test is used to evaluate how the final results would have changed if the set case study considered for validation were different by using the Jackknife approach. In particular, a one-by-one removal of the 41 urban districts is performed and every removal the MP problem is solved to provides the vector of weights ***v***. Finally, a statistical variation of the vector ***v*** is obtained on the basis of the resampling of input data.

Secondly, an additional test identifies the *Absolute Error* and the *Mean Relative Error* of ${{I}_{UHII}}^{Ud}({\varvec{v}})$ in comparison with the effective absolute max UHII obtained from the bibliography. Also in this case, a Jackknife resampling is used to obtain the statistical variation of the absolute for every one-by-one removal of the 41 urban districts.

Thirdly, the *Pearson correlation coefficient* is evaluated to measure the linear correlation between the criteria and the UHII. This analysis allows understanding the presence of a predictive relationship between the considered *parameters* and the investigated phenomenon. In particular, the absence of low and null correlation coefficients can justify the presence of all the eleven parameters in both the AHP framework and the index evaluation.

## Supplementary information

Supplementary Figures.

Supplementary Methodology.

Supplementary Tables.
