# Supplementary material for: Development of a holistic urban heat island evaluation methodology
Source: Sci Rep. 2020 Oct 21;10:17913. doi: 10.1038/s41598-020-75018-4 (PMC7578064; doi:10.1038/s41598-020-75018-4)
Supplement: Supplementary file 1 — Supplementary Figures. [file 41598_2020_75018_MOESM1_ESM.pdf]

Development of a holistic urban heat island evaluation methodology

Valentino Sangiorgio[1*], Francesco Fiorito[1,2], Mattheos Santamouris [2]
[1] *DICATECH, Politecnico di Bari, Via Edoardo Orabona 4, Bari, Italy*
[2] *High Performance Architecture, School of Built Environment, University of New South Wales, Sydney, NSW, 2052, Australia.*
*valentino.sangiorgio@poliba.it

This document contains **Supplementary Figures:**

Supplementary Figure S1,
Supplementary Figure S2,
Supplementary Figure S3.

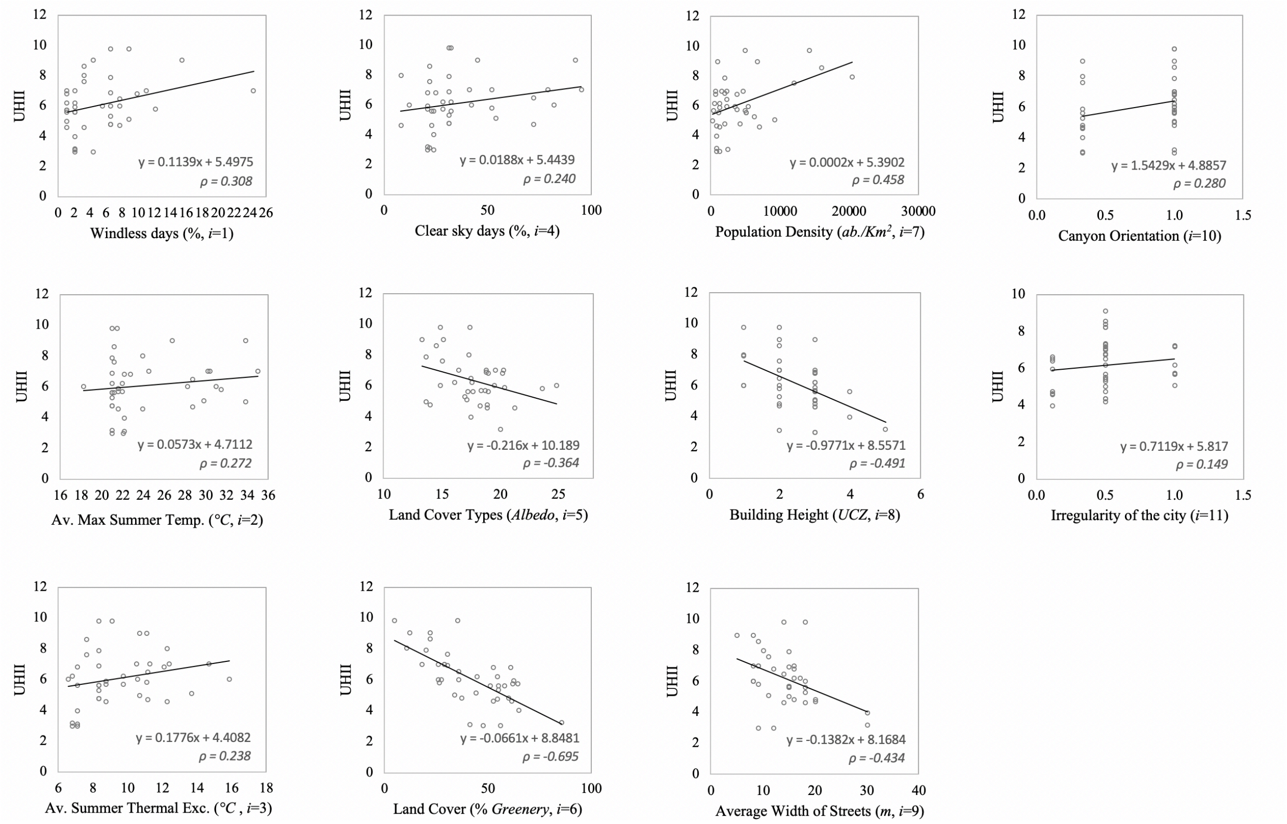

Fig S1. Scatter plots and corresponding *Pearson correlation coefficient* (*ρ*) evaluated to measures the linear correlation between the defined eleven criteria *i* (with *i*=1, …, 11) and the absolute max UHII. The figure was created by using Matlab R2016a v9.0.0.341360 (https://www.mathworks.com), and Microsoft Excel v16.33 (https://www.microsoft.com).

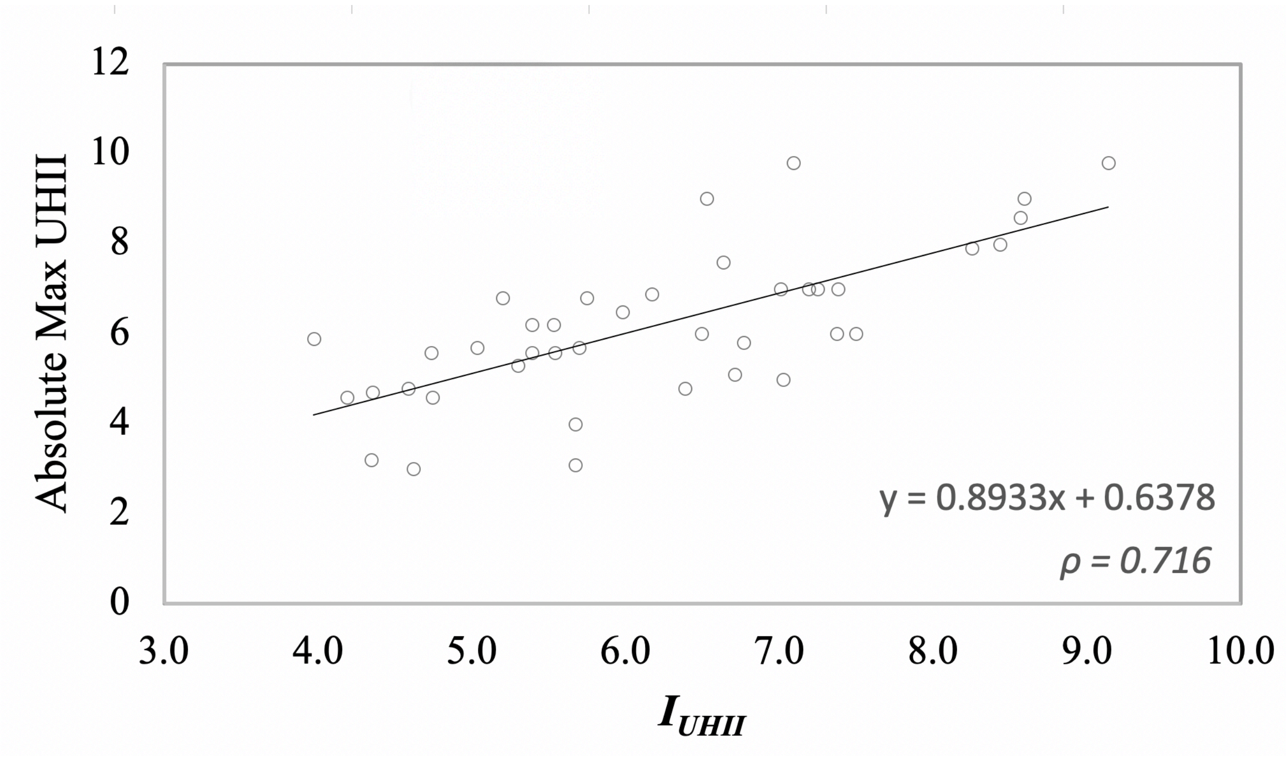

Fig S2. Scatter plots and corresponding *Pearson correlation coefficient* (ρ) evaluated to measures the linear correlation between the proposed index $\boldsymbol{I_{UHII}}$ and the absolute max UHII. The figure was created by using Matlab R2016a v9.0.0.341360 (https://www.mathworks.com), and Microsoft Excel v16.33 (https://www.microsoft.com).

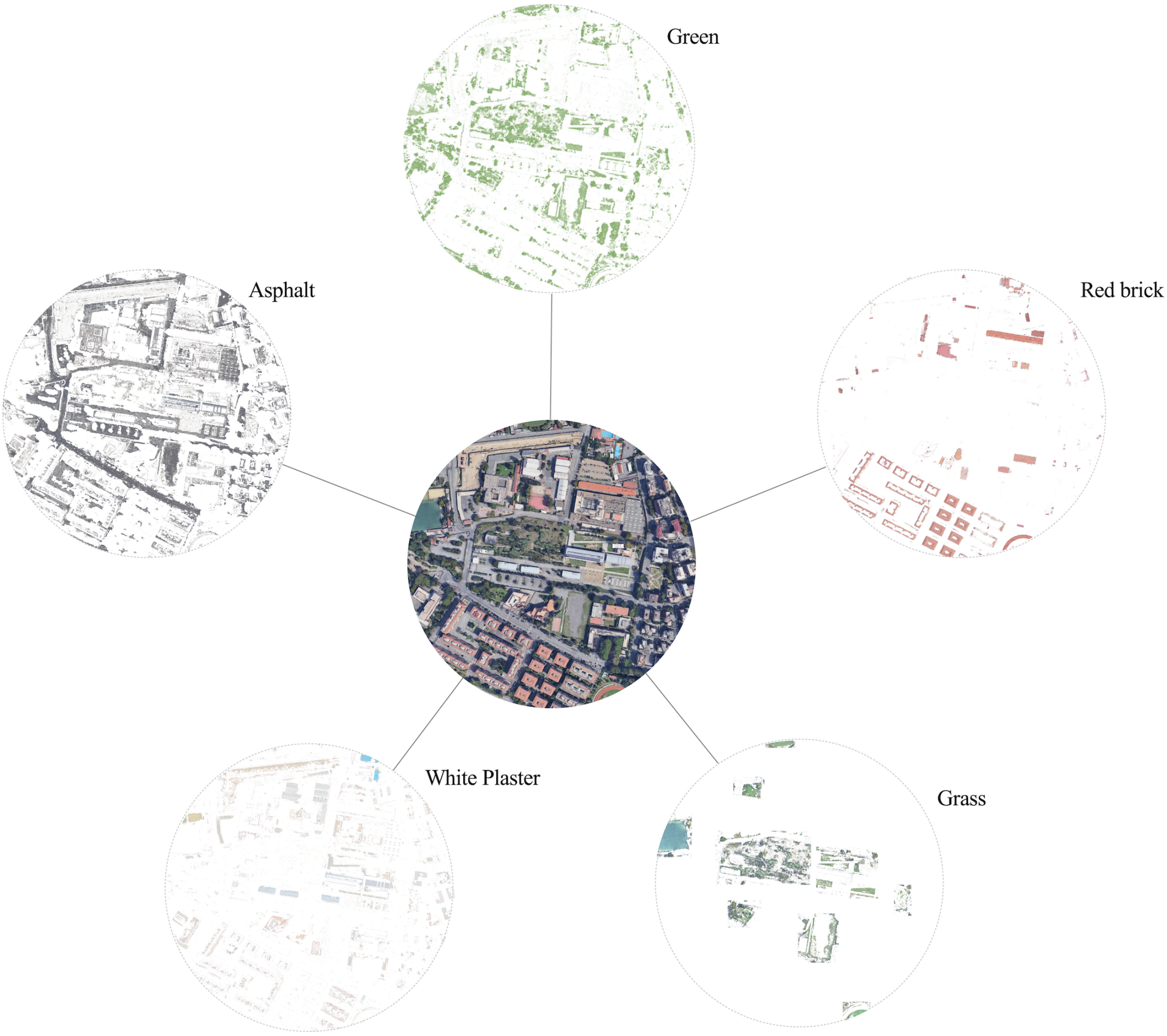

Fig S3. Example of a part of the image analysis to extract data of the characteristics of the city (*Ud*=32, Rome, Roma3, Italy). The figure was created by using Matlab R2016a v9.0.0.341360 (https://www.mathworks.com) and Adobe Photoshop 2020 v21.1.0 (https://www.adobe.com).
