# Supplementary material for: Development of a holistic urban heat island evaluation methodology
Source: Sci Rep. 2020 Oct 21;10:17913. doi: 10.1038/s41598-020-75018-4 (PMC7578064; doi:10.1038/s41598-020-75018-4)
Supplement: Supplementary file 2 — Supplementary Methodology. [file 41598_2020_75018_MOESM2_ESM.docx]

**Development of a holistic urban heat island evaluation methodology**

Valentino Sangiorgio^1*^, Francesco Fiorito^1,2^, Mattheos Santamouris ^2^

^1^ *DICATECH, Politecnico di Bari, Via Edoardo Orabona 4, Bari, Italy*

^2^ *High Performance Architecture, School of Built Environment, University of New South Wales, Sydney, NSW, 2052, Australia.*

*valentino.sangiorgio@poliba.it

This document contains the **Supplementary methodology**

**Supplementary methodology** includes four subsections:

“The AHP to obtain a synthetic index”,

“AHP step1: The structuring the problem of the UHI phenomenon”,

“AHP step2: preliminary weights evaluation”,

and “AHP step3: definition of the index”

Supplementary Methodology

This section recalls the standard methodological approach of AHP1 as is described in detail in Sangiorgio et al.^1^. In addition, this approach is applied to structure the problem of the UHI phenomenon, obtain a preliminary weights evaluation, and define the index ***I_UHII._***

The AHP to obtain a synthetic index

Starting from a decision problem, the first step consists of *structuring the problem* according to a hierarchical scheme, to provide a detailed, simple and systematic decomposition of the problem into its basic parameters. First, it is necessary to identify the main AHP goal and the related macro-criteria, criteria and intensity ranges to achieve the goal.

The second is the *weight evaluation* and provides the weights that are necessary to define the index. Considering *n* ordered criteria of comparison (i.e. criteria or intensity ranges), a *n×n judgments matrix A* is defined, where each upper diagonal element *a_ij_*>0 is generated by comparing the *i*^-th^ with the *j*^-th^ element through the fundamental scale of absolute numbers (Table S4). This semantic scale is composed of verbal scales that are associated with numerical values (1, 3, 5, 7, 9) and compromises (1.5, 2, 4, 6, 8) between them.

The principal eigenvalue method is used by the AHP in order to derive ratio scale priority vectors from *judgments matrices*. In particular, the following eigenvector problem is solved to obtain the weights:

*A w = λ_max_ w*  (7)

where *w* is the eigenvector and *λ_max_* is the principal eigenvalue. Moreover, the coherence of the assigned judgment is defined by the consistency index *CI* according to Saaty^2^. The index increases proportionally with the incoherence of the matrix:

$$CI=\frac{\lambda_{max}-n}{n-1}. (8)$$

**Supplementary Methodology Table S4 .** Saaty’s Fundamental Scale^2^

| ***a*_ij_** | **Verbal scale** |
| --- | --- |
| *a_ij_* = 1 | Equal importance |
| *a_ij_* = 3 | Moderate importance of one over another |
| *a_ij_* = 5 | Strong importance |
| *a_ij_* = 7 | Very strong importance |
| *a_ij_* = 9 | Extreme importance |
| 1.5 - 4 - 6 - 8 | Intermediate value |
| 1/9,1/8,….,1/2 | The reciprocal expresses an opposite judgment |

Operationally, in order to verify the coherence of the paired comparisons, a consistency test is executed by the *Consistency Ratio* (*CR*). In particular, *CR* is obtained by considering the ratio between *CI* and its expected value, denoted by the Random Index (*RI*). The values of *RI* is determined by considering a large number of positive reciprocal matrices of order *n* whose entries are randomly chosen in the set of values *n* ∈ {1,2,..., 11}. The following relationship holds:


$$CR=\frac{CI}{RI\left( n \right)}. (9)$$

In the related literature the different values of *RI* are proposed. In this work, those determined in Noble and Sanchez^3^ were used, as reported in Table S5. On the basis of several empirical studies, Saaty concluded that the value of Consistency Ratio CR< 0.10 is acceptable^2^.

The third step, i.e., the *summary of priority,* is performed to define the index and specify the use of the obtained weights in the index definition. In particular, the weights of the alternatives is combined with the weights of each criterion. The index equation is obtained by multiplying each criteria weight by the alternative weight and totalling the results for each alternative.

**Supplementary Methodology Table S5.** Noble’s Random Consistency Index^3^

| **n** | **1** | **2** | **3** | **4** | **5** | **6** | **7** | **8** | **9** | **10** | **11** |
| --- | --- | --- | --- | --- | --- | --- | --- | --- | --- | --- | --- |
| R.I. | *0* | *0* | *0.49* | *0.82* | *1.03* | *1.16* | *1.25* | *1.31* | *1.36* | *1.39* | *1.42* |

AHP step1: The structuring the problem of the UHI phenomenon.

The first step in AHP consists in the *Structure of the Problem*.

Eleven *criteria i* (with *i*=1,…,11) are defined and grouped into four *macro-criteria*. For each *criterion* a set of intensity ranges *j* (with *j*=1,…,*n_i_*) is defined to characterize its intensity levels. The structuring of the problem is discussed as follows:

The First *macro-criterion* regards the *Meteorological Variables* in which the first three criteria are grouped:

1. *Windless Days* is a determining factor in the UHI phenomenon. Indeed, it is widely accepted in the related literature^4^ that UHI is higher between few hours after sunset in the ideal condition of no wind^5,6,7^. Stronger winds modify the cooling rates in the urban and rural zones and alter the magnitude of urban heat island^8^. The wind speed start to be effective in reducing the UHI effect with a speed greater than 5 km/h^9^. In addition, the study of Klysik and Fortuniak^10^ reports that when the city wind speed is about 7.2 km/h during day and 14.4 km/h during the night there is a decrease of UHII but the phenomenon could still be observed. Finally, when the critical wind speed overcome 20 km/h or 25 km/h the UHII could vanish. Consequently, in order to consider the possible occurrence of the ideal “low wind” conditions, the number of days in which the average wind speed is below 5 km/h are considered. In addition, this number of days is expressed as a percentage of the total days of the considered summer period. Five different intensity ranges are defined (*n_1_*=5) as: i) less than 1%, ii) 1%-5 %, iii) 5%-10% , iv) 10%-15%, v) and more than 15%.
2. *Average Max Summer Temperature* influences the UHI intensities as demonstrated in the studies of Santamouris^11,12^ involving different European cities. The increase of the phenomenon become critical by average max summer temperatures higher than 28°C. By exploiting the studies of Santamouris ^11,12^ and New et al.^13^ regarding high-resolution data set of surface climate over global land areas six intensity ranges are defined (*n_2_*=6): i) more than 30°C, ii) 28-30°C, iii) 26-28°C, iv) 24-26°C, v) 22-24°C, vi) less than 22°C.
3. *Average Summer Thermal Excursion* can influence the UHI intensities. This criterion considers the average of the difference between maximum and minimum temperatures evaluated for each day of the summer period. The increase of the phenomenon becomes critical by thermal excursion higher than 16°C^14^. To consider this criterion, six intensity ranges are defined by considering intervals that can effectively classify the various climatic typologies of cities (*n_3_*=6): i) more than 16°C, ii) 14-16°C, iii) 12-14°C, iv) 10-12°C, v) 8-10°C, vi) less than 8°C.
4. *Clear sky days* also have an important influence and are straight connected to the direct solar irradiation. In particular, clouds do not allow the terrestrial infrared radiation to escape, absorb and reemit it back altering the thermal balance of the area^15^. Consequently, in order to consider the possible occurrence of the ideal “no cloud” condition, the number of days in which the sky is clear (1 or 2 Okta) is considered. In addition, this number of days is expressed as a percentage of the total days of the considered summer period. Eight different Intensity Ranges are determined (*n_4_*=8): varying from less than 10%, and more than 70%.

The Second *macro-criterion* takes the *Characteristics of the City (Albedo)* into account to evaluate the UHII. It considers the two phenomena of evapotranspiration of green areas and the absorption of solar radiation due to the city's material. To this aim, the fifth and sixth criteria are defined:

5) *Land Cover Types (Albedo)* it is an important factor to consider the ability of surfaces to reflect solar radiation in creating UHI^16^. Light-coloured surfaces return a large part of the sunrays back to the atmosphere (high albedo). Dark surfaces absorb the rays from the sun (low albedo)^17^. The average of city albedo can be evaluated by considering the land cover types through satellite photos. The relation between the type of surface and the index of albedo is established by exploiting the correlation between albedo and land cover types of Bradley et al.^18^. In addition, ten intensity ranges (*n_5_*=10) are defined ranging between 0.12 and 0.30.

6) *Land Cover (Greenery)* is taken into account to consider the phenomenon of evapotranspiration. Several authors studied the related cooling effect given by the combination of evaporation and^19,20^ in order to exploit this effect to mitigate the UHII. In this work, ten ranges (*n_6_*=10) are defined to quantify the percentage of the city form less than 5% to more than 45%, with differences of 5% between two consecutive ranges.

Another significant *macro-criterion* is the Anthropogenic Heat that can be considered with the following criterion:

7) *Population Density* could have twofold effects on heat generation. A direct effect related to the high number of metabolisms (more people) and an indirect effect connected with the number of vehicles, factories and heat producer utilities^21^. In addition, a study of Merkin ^22^ show a linear dependence between anthropogenic heat and population density. The related ranges (*n_7_*=11) can be defined basing on the number of inhabitants per square kilometre varying from form less than 1,000 ab/Km^2^ to more than 18,000 ab/Km^2^.

The final macro-criterion considers the effects caused by the *City Canyons*. To this aim, four *criteria* can be defined on the basis of the related literature and are discussed in the following:

8) *Buildings Heights* interferes with air flows. In particular the height obstacles, in this case the buildings, directly influence the potential creation of the UHI^23^. Five ranges (*n_8_*=5) of building height are defined by following the classification of Oke^24^ including the following five Urban Climate Zones (UCZ): class 1 Intensely developed high-rise building, class 2 Intensely developed 2-5 storey building, class 3 Highly developed dose set houses, class 4 Highly developed low building, class 5 Medium developed 1 or 2 storey houses

9) *Width of street* is another geometrical criterion influencing the *City Canyons* effect on the UHI^22^. Analogously to criterion nine, five ranges (*n_9_*=5) of average width of street are defined and ranging from less than 5m to more than 25m.

10) *Canyons orientation,* referring to the cardinal points, influences the quantity of direct radiation into the air volume, contributing to the amount of absorbed solar radiation of the city^25^. Four different *intensity ranges* (*n_10_*=4) are selected by considering the possible orientation of the principal canyon of the urban districts with respect to the cardinal points.

11) *Irregularity of the city,* is the last criterion which affects the UHI phenomenon. Statistical analysis indicates the importance of geometry of street canyons to reduce street ventilation in the city improving the UHI^25^. The three possible intensity ranges (*n_11_*=3) are defined as irregular, mixed, or regular street arrangement.

AHP step2: preliminary weights evaluation.

The second step of the AHP is devoted to the weights evaluation. The weights of *criteria* and *intensity ranges* are defined as follows in relation with the defined *Structure of the Problem*:

- *v_i_* is the weight associated with each *i*^-th^ *criterion*

- *w_ij_* is the weight associated with each *j*^-th^ *intensity range* related to the *i*^-th^ criterion

The weight calculations of the *intensity ranges* can exploit both quantitative and qualitative data. To provide an example, the *Windless Days* and to the *Irregularity of the City* criteria are shown.

The weights of the intensity ranges associated to the *Windless Days* are obtained by assuming a linear evolution of cooling effect in relation with the “wind days” decrease^15^. Pairwise comparisons of the alternatives are carried out performing a quantitative analysis to achieve the *judgment matrix A_1_*. The weights are obtained by solving the eigenvector problem for matrix *A_1_*.

**Table S6**. Judgment Matrix *A_1_,* weights*,* and *CR* obtained for the *intensity ranges* related to “*Average Wind Speed*” criterion

| ***A_1_*** | **(a)** | **(b)** | **(c)** | **(d)** | **(e)** | ***CR*** | ***w_1,j_*** |
| --- | --- | --- | --- | --- | --- | --- | --- |
| More than 15% | 1.0 | 1.4 | 2.3 | 7.0 | 35.0 | 0.002 | 1.00 |
| 10% -15% | 0.7 | 1.0 | 1.7 | 5.0 | 25.0 |  | 0.71 |
| 5% - 10% | 0.4 | 0.6 | 1.0 | 3.0 | 15.0 |  | 0.43 |
| 1% - 5% | 0.1 | 0.2 | 0.3 | 1.0 | 5.0 |  | 0.14 |
| Less than 1% | 0.0 | 0.0 | 0.1 | 0.2 | 1.0 |  | 0.03 |

The resulting matrix satisfied the Consistency Ratio requirement *CR*<0.1 and derived consistent weights *w_1j_*, normalized between 0 to 1 (see Table S6).

The weights of the intensity ranges associated to the *Irregularity of the City*, are obtained by the matrix *A_11_* exploiting a qualitative evaluation. This criterion considers the increase of UHI phenomenon due to the irregularity of the city which affects the street ventilation. The matrix is obtained by considering a maximum increase of UHII when streets are irregular and a minimum when the arrangement of the street is regular. By perform the pairwise comparison ed exploiting Table S4, *A_11_* is obtained.

**Table S7.** Judgment Matrix *A_11_,* weights*,* and *CR* obtained for the *intensity ranges* related to *Irregularity of the City*

| ***A_11_*** | **(i.s.)** | **(m.s.)** | **(r.s.)** | ***CR*** | ***w_11,j_*** |
| --- | --- | --- | --- | --- | --- |
| Irregular Streets (i.s.) | 1.0 | 2.0 | 9.0 | 0.001 | 1.00 |
| Mixed Streets (m.s.) | 0.5 | 1.0 | 4.5 |  | 0.50 |
| Regular Streets (r.s.) | 0.1 | 0.2 | 1.0 |  | 0.11 |

The weights of matrix *A_11_* are derived by solving the eigenvector problem described in supplementary methodology. The resulting matrix satisfies the Consistency Ratio requirement *CR*<0.1 and derives consistent weights *w_11,j_* (normalized to 1), as shown in Table S7. After obtaining the weights of the *intensity ranges* related to each criterion, the second AHP step obtained the tabulated weights related to the *Structure of the Problem*.

AHP step3: definition of the index.

After weighting, the potential Urban Heat Island Intensity Index (***I_UHII_***) can be defined. This operation coincides with the third step of the summary of priority. The formula is obtained by multiplying each *criteria* weight by the *intensity range* weight and adding the results, as in the classical AHP procedure:

References:

1. Sangiorgio, V., Uva, G., Fatiguso, F., & Adam, J. M. A new index to evaluate exposure and potential damage to RC building structures in coastal areas. *Engineering failure analysis*. **100**, 439-455 (2019).

2. Saaty, T. L. Decision making with the analytic hierarchy process. *International journal of services sciences*. **1**, 83-98 (2008).

3. Noble, E. E., & Sanchez, P. P. A note on the information content of a consistent pairwise comparison judgment matrix of an AHP decision maker. *Theory and Decision*. **34**, 99-108 (1993).

4. Rizwan, A. M., Dennis, L. Y., & Chunho, L. I. U. A review on the generation, determination and mitigation of Urban Heat Island. *Journal of Environmental Sciences*. **20**, 120-128 (2008).

5. Erell, E., & Williamson, T. Intra‐urban differences in canopy layer air temperature at a mid‐latitude city. *International Journal of Climatology: A Journal of the Royal Meteorological Society*. **27**, 1243-1255 (2007).

6. Eliasson, I. Intra-urban nocturnal temperature differences: a multivariate approach. *Climate Research*. **7**, 21-30 (1996).

7. Papanikolaou, N. M., Livada, I., Santamouris, M., & Niachou, K. The influence of wind speed on heat island phenomena in Athens, Greece. *International Journal of Ventilation*. **6**, 337-348 (2008).

8. Tzavali, A., Paravantis, J. P., Mihalakakou, G., Fotiadi, A., & Stigka, E. Urban heat island intensity: a literature review. *Fresenius Environmental Bulletin*. **24**, 4537-4554 (2015).

9. Kim, Y. H., & Baik, J. J. Maximum urban heat island intensity in Seoul. *Journal of applied meteorology*. **41**, 651-659 (2002).

10. Kłysik, K., & Fortuniak, K. Temporal and spatial characteristics of the urban heat island of Łódź, Poland. *Atmospheric environment*. **33**, 3885-3895 (1999).

11. Santamouris, M. Cooling the cities–a review of reflective and green roof mitigation technologies to fight heat island and improve comfort in urban environments. *Solar Energy*. **103**, 682–703 (2014).

12. Santamouris, M. On the energy impact of urban heat island and global warming on buildings. *Energy and Buildings.* **82**,100–113 (2014).

13 New, M., Lister, D., Hulme, M., & Makin, I. A high-resolution data set of surface climate over global land areas. *Climate research*. **21**, 1-25 (2002).

14. Bevilacqua, P., Mazzeo, D., Bruno, R., & Arcuri, N. Surface temperature analysis of an extensive green roof for the mitigation of urban heat island in southern mediterranean climate. *Energy and Buildings*. **150**, 318-327 (2017).

15. Morris, C. J. G., Simmonds, I., & Plummer, N. Quantification of the influences of wind and cloud on the nocturnal urban heat island of a large city. *Journal of Applied Meteorology*. **40**, 169-182 (2001).

16. Giridharan R, Ganesan S, Lau S. S. Y. Daytime urban heat island effect in high-rise and high-density residential developments in Hong Kong. *Energy and Buildings*. **36**, 525-534 (2004).

17. Taha H, Konopacki S, Gabersek S. Impacts of large scale modifications on meteorological conditions and energy use: A 10-region modeling study. *Theoretical and Applied Climatology*. **62**, 175–185 (1999).

18. Bradley, A. V., Thornes, J. E., Chapman, L., Unwin, D., & Roy, M. Modelling spatial and temporal road thermal climatology in rural and urban areas using a GIS. *Climate Research*. **22**, 41-55 (2002).

19. Honjo, T., & Takakura, T. Simulation of thermal effects of urban green areas on their surrounding areas. Energy and buildings. **15**, 443-446 (1990).

20. Gómez, F., Gil, L., & Jabaloyes, J. Experimental investigation on the thermal comfort in the city: relationship with the green areas, interaction with the urban microclimate. *Building and Environment*. **39**, 1077-1086 (2004).

21. Hung T., Uchihama D., Ochi S., Yasuoka Y. Assessment with satellite data of the urban heat island effects in Asian mega cities. International *Journal of Applied Earth Observation and Geo-Information*. **8**, 34-48 (2006).

22. Merkin, R. The Urban Heat Island's Effect on the diurnal temperature range. Doctoral dissertation, Massachusetts Institute of Technology (2004).

23. Arnfield, A. J., & Grimmond, C. S. B. An urban canyon energy budget model and its application to urban storage heat flux modeling. Energy and buildings. **27**, 61-68 (1998).

24. Oke, T.R. Initial guidance to obtain representative meteorological observations at urban sites. Instruments and observing methods, no. 81. Canada. World Meteorological Organization. (2006).

25. Unger, J. Connection between urban heat island and sky view factor approximated by a software tool on a 3D urban database. *International Journal of Environment and Pollution*. **36**, 59-80 (2009).
