# Supplementary material for: Development of a holistic urban heat island evaluation methodology
Source: Sci Rep. 2020 Oct 21;10:17913. doi: 10.1038/s41598-020-75018-4 (PMC7578064; doi:10.1038/s41598-020-75018-4)
Supplement: Supplementary file 3 — Supplementary Tables. [file 41598_2020_75018_MOESM3_ESM.pdf]

# Development of a holistic urban heat island evaluation methodology

Valentino Sangiorgio[1*], Francesco Fiorito[1,2], Mattheos Santamouris [2]

[1] *DICATECH, Politecnico di Bari, Via Edoardo Orabona 4, Bari, Italy*

[2] *High Performance Architecture, School of Built Environment, University of New South Wales, Sydney, NSW, 2052, Australia.*

*valentino.sangiorgio@poliba.it

This document contains the following **Supplementary Tables:**

**Supplementary Tables:**

**Table S1,**
**Table S2.**

**Table S1.** Tabulated weight obtained by applying AHP Step 2

| **Macro-Criteria** | ***Criteria*** | $v_i$ | **Value** | ***Intensity ranges*** | $w_{i,j}$ | **Value** |
|---|---|---|---|---|---|---|
| **Meteorological Variables** | Windless Days | $v_1$ | 0.21 | More than 15% | $w_{1,1}$ | 1.00 |
| | | | | 10% -15% | $w_{1,2}$ | 0.71 |
| | | | | 5% - 10% | $w_{1,3}$ | 0.43 |
| | | | | 1% - 5% | $w_{1,4}$ | 0.14 |
| | | | | Less than 1% | $w_{1,5}$ | 0.03 |
| | Average Max Summer Temperature | $v_2$ | 0.10 | More than 30°C | $w_{2,1}$ | 1 |
| | | | | 28°C - 30°C | $w_{2,2}$ | 0.85 |
| | | | | 26°C - 28°C | $w_{2,3}$ | 0.7 |
| | | | | 24°C - 26°C | $w_{2,4}$ | 0.55 |
| | | | | 22°C - 24°C | $w_{2,5}$ | 0.4 |
| | | | | Less than 22°C | $w_{2,6}$ | 0.25 |
| | Average Summer Thermal Excursion | $v_3$ | 0.10 | More than 16°C | $w_{3,1}$ | 1 |
| | | | | 14°C - 16°C | $w_{3,2}$ | 0.85 |
| | | | | 12°C - 14°C | $w_{3,3}$ | 0.7 |
| | | | | 10°C -12°C | $w_{3,4}$ | 0.55 |
| | | | | 8°C - 10°C | $w_{3,5}$ | 0.4 |
| | | | | Less than 8°C | $w_{3,6}$ | 0.25 |
| | Clear Sky Days | $v_4$ | 0.33 | More than 70% | $w_{4,1}$ | 1.00 |
| | | | | 60% -70% | $w_{4,2}$ | 0.87 |
| | | | | 50% -60% | $w_{4,3}$ | 0.73 |
| | | | | 40% -50% | $w_{4,4}$ | 0.60 |
| | | | | 30% -40% | $w_{4,5}$ | 0.47 |
| | | | | 20% -30% | $w_{4,6}$ | 0.33 |
| | | | | 10% -20% | $w_{4,7}$ | 0.20 |
| | | | | Less than 10% | $w_{4,8}$ | 0.07 |
| **Characteristics of the City** | Land Cover Types (Albedo) | $v_5$ | 3.47 | 0.10 - 0.12 | $w_{5,1}$ | 1 |
| | | | | 0.12 - 0.14 | $w_{5,2}$ | 0.85 |
| | | | | 0.14 - 0.16 | $w_{5,3}$ | 0.73 |
| | | | | 0.16 - 0.18 | $w_{5,4}$ | 0.65 |
| | | | | 0.18 - 0.20 | $w_{5,5}$ | 0.58 |
| | | | | 0.20 - 0.22 | $w_{5,6}$ | 0.52 |
| | | | | 0.22 - 0.24 | $w_{5,7}$ | 0.48 |
| | | | | 0.24 - 0.26 | $w_{5,8}$ | 0.44 |
| | | | | 0.26 - 0.28 | $w_{5,9}$ | 0.41 |
| | | | | 0.28 - 0.30 | $w_{5,10}$ | 0.38 |
| | Land Cover (% Greenery) | $v_6$ | 2.62 | Less than 5% | $w_{6,1}$ | 1 |
| | | | | 5 - 10% | $w_{6,2}$ | 0.9 |
| | | | | 10 - 15% | $w_{6,3}$ | 0.8 |
| | | | | 15 - 20% | $w_{6,4}$ | 0.7 |
| | | | | 20 - 25% | $w_{6,5}$ | 0.6 |
| | | | | 25 - 30% | $w_{6,6}$ | 0.5 |
| | | | | 30 - 35% | $w_{6,7}$ | 0.4 |
| | | | | 35 - 40% | $w_{6,8}$ | 0.3 |
| | | | | 40 - 45% | $w_{6,9}$ | 0.2 |
| | | | | More than 45% | $w_{6,10}$ | 0.1 |
| **Anthropogenic Heat** | Population Density | $v_8$ | 0.75 | More than 20,000 ab/Km$^2$ | $w_{7,1}$ | 1 |
| | | | | 18,000-20,000 ab/Km$^2$ | $w_{7,2}$ | 0.91 |
| | | | | 16,000-18,000 ab/Km$^2$ | $w_{7,3}$ | 0.82 |
| | | | | 14,000-16,000 ab/Km$^2$ | $w_{7,4}$ | 0.73 |
| | | | | 12,000-14,000 ab/Km$^2$ | $w_{7,5}$ | 0.64 |
| | | | | 10,000-12,000 ab/Km$^2$ | $w_{7,6}$ | 0.55 |
| | | | | 8,000-10,000 ab/Km$^2$ | $w_{7,7}$ | 0.46 |
| | | | | 6,000-8,000 ab/Km$^2$ | $w_{7,8}$ | 0.36 |
| | | | | 4,000-6,000 ab/Km$^2$ | $w_{7,9}$ | 0.26 |
| | | | | 2,000-4,000 ab/Km$^2$ | $w_{7,10}$ | 0.15 |
| | | | | Less than 2,000 ab/Km$^2$ | $w_{7,11}$ | 0.07 |
| **City canyon** | Building Height (UCZ) | $v_9$ | 0.99 | More than 20m | $w_{8,1}$ | 1 |
| | | | | 15 - 20 m | $w_{8,2}$ | 0.5 |
| | | | | 10 - 15 m | $w_{8,3}$ | 0.38 |
| | | | | 5 - 10 m | $w_{8,4}$ | 0.25 |
| | | | | Less than 5m | $w_{8,5}$ | 0.13 |
| | Width of Streets | $v_{10}$ | 1.99 | Less than 2m | $w_{9,1}$ | 1 |
| | | | | 2 - 3 m | $w_{9,2}$ | 0.85 |
| | | | | 3 - 4 m | $w_{9,3}$ | 0.69 |
| | | | | 4 - 5 m | $w_{9,4}$ | 0.54 |
| | | | | More than 5 m | $w_{9,5}$ | 0.23 |
| | Canyon Orientation | $v_{11}$ | 1.01 | South | $w_{10,1}$ | 1 |
| | | | | South - West | $w_{10,2}$ | 0.33 |
| | | | | South - East | $w_{10,3}$ | 0.33 |
| | | | | East | $w_{10,4}$ | 0.11 |
| | Irregularity of the city | $v_{12}$ | 0.61 | Irregular street arrangement | $w_{11,1}$ | 1 |
| | | | | Mixed street arrangement | $w_{11,2}$ | 0.5 |
| | | | | Regular street arrangement | $w_{11,3}$ | 0.11 |

**Table S2.** Complete dataset for validation and calibration.

| | Standard fixed measuring station | | | Rural Station | | | | | | Urban districts | Data from bibliography | Tutiempo database, Copernicus data, Clara database and Weatherspark database | | | | Data processing - satellite imagery | | Eurostat dataset | Satellite imagery - Google Maps and Technical cartographies (when available) | | | |
|---|---|---|---|---|---|---|---|---|---|---|---|---|---|---|---|---|---|---|---|---|---|---|
| | City | Acquisition period | Geographic coordinates | Location | Geographic coordinates | Location | Geographic coordinates | Location | Geographic coordinates | *Ud* | Max UHII | Windless days (%) | Average Max Summer Temperature (°C) | Average Spring Thermal Excursion (°C) | Clear sky days (%) | Land Cover Types (Albedo) | Land Cover (% Greenery) | Population Density (ab./Km2) | Building Height (UCZ)* | Average Width of Streets (m) | Canyon Orientation | Irregularity of the city |
| Data for Calibration | Adana | 1980 - 1990 | 36°58'48.0"N 35°18'00.0"E | Islahiye | 37°01'12.0"N 36°22'48.0"E | | | | | 1 | 9.0 | 15.6 | 33.8 | 10.7 | 92.0 | 13.3 | 12.1 | 909.4 | 3 | 5.1 | South | Regular |
| | Apeldoorn | 01/2008 - 06/2009 | 52°12'00.0"N 5°55'58.8"E | Deelen | 52°04'45.5"N 5°56'51.3"E | | | | | 2 | 6.2 | 1.1 | 22.0 | 9.7 | 28.0 | 16.1 | 44.6 | 458.3 | 3 | 17.1 | South | Mixed |
| | Assen | 01/2007 - 03/2009 | 53°00'36.0"N 6°34'04.8"E | Eelde | 53°07'38.0"N 6°35'16.2"E | | | | | 3 | 4.0 | 2.2 | 22.2 | 7.1 | 24.0 | 17.5 | 65.0 | 813.37 | 4 | 30.1 | South - East | Irregular |
| | Bucharest | Summer 2007 | 44°24'56.9"N 26°05'30.0"E | Afumati | 44°31'46.5"N 26°15'13.2"E | | | | | 4 | 5.1 | 8.9 | 29.8 | 13.7 | 54.0 | 17.1 | 44.3 | 9225.2 | 3 | 11.1 | South | Mixed |
| | Bursa | 1980 - 1990 | 40°10'48.0"N 29°04'12.0"E | Canakkale | 40°08'02.3"N 26°25'41.8"E | Bandırma | 40°19'13.3"N 27°58'14.9"E | Bilecik | 40°09'00.0"N 29°58'12.0"E | 5 | 7.0 | 2.2 | 30.4 | 10.5 | 79.0 | 18.8 | 25.9 | 1508.5 | 2 | 9.1 | South | Irregular |
| | Damwoude | 01/2005 - 04/2009 | 53°17'27.6"N 5°58'40.8"E | Leeuwarden Airport | 53°13'09.7"N 5°45'15.8"E | | | | | 6 | 3.2 | 2.2 | 21.0 | 6.8 | 21.0 | 20.0 | 85.8 | 800.0 | 5 | 30.1 | South | Mixed |
| | Delft | 01/2007 - 03/2009 | 51°58'48.0"N 4°20'24.0"E | Rotterdam Airport | 51°57'23.3"N 4°26'35.3"E | | | | | 7 | 4.8 | 6.7 | 21.0 | 8.3 | 31.0 | 18.9 | 59.8 | 4211.0 | 3 | 16.1 | South - East | Regular |
| | Doornenburg | 01/2009 - 06/2009 | 51°53'24.0"N 6°00'00.0"E | Deelen | 52°04'45.5"N 5°56'51.3"E | | | | | 8 | 5.7 | 1.1 | 22.0 | 9.7 | 28.0 | 18.7 | 64.5 | 338.6 | 3 | 15.1 | South | Mixed |
| | Firenze | Summer 2002 | 43°46'28.1"N 11°15'17.7"E | Firenze countryside | 43°44'09.2"N 11°15'34.8"E | | | | | 9 | 5.8 | 12.2 | 31.5 | 11.1 | 52.0 | 23.6 | 26.4 | 3709.6 | 2 | 9.1 | South | Mixed |
| | Gaziantep | 1980 - 1990 | 37°04'48.0"N 37°22'12.0"E | Islahiye | 37°01'12.0"N 36°22'48.0"E | | | | | 10 | 5.0 | 1.1 | 33.8 | 10.7 | 94.0 | 13.6 | 34.1 | 247.3 | 3 | 15.1 | South | Mixed |
| | Glasgow | Spring/Summer 2011 | 55°51'32.0"N 4°15'25.7"W | Cochno Farm | 55°56'24.3"N 4°24'38.5"W | | | | | 11 | 6.0 | 6.7 | 18.2 | 6.6 | 12.0 | 14.9 | 25.8 | 3538.6 | 1 | 18.1 | South | Regular |
| | Groningen | 01/1999 - 03/2009 | 53°12'57.6"N 6°34'01.2"E | Eelde | 53°07'38.0"N 6°35'16.2"E | | | | | 12 | 3.1 | 2.2 | 22.2 | 7.1 | 22.0 | 15.7 | 41.1 | 2416.7 | 2 | 10.1 | South - East | Mixed |
| | Haarlem | 12/2005 - 02/2008 | 52°22'12.0"N 4°39'36.0"E | Schiphol | 52°18'25.1"N 4°45'39.2"E | | | | | 13 | 5.7 | 1.1 | 21.5 | 8.7 | 21.0 | 18.3 | 62.0 | 4960.8 | 3 | 15.1 | South | Irregular |
| | Heemskerk | 01/2005 - 12/2005 | 52°29'56.4"N 4°40'58.8"E | Schiphol | 52°18'25.1"N 4°45'39.2"E | | | | | 14 | 5.9 | 2.2 | 21.5 | 8.7 | 21.0 | 20.4 | 62.6 | 1234.1 | 3 | 18.1 | South - West | Regular |
| | Heerhugowaard | 01/2005 - 04/2009 | 52°40'12.0"N 4°50'49.2"E | Berkhout | 52°38'26.6"N 4°59'51.2"E | | | | | 15 | 6.2 | 2.2 | 21.0 | 6.8 | 32.0 | 17.6 | 54.7 | 1282.3 | 3 | 16.1 | South | Mixed |
| | Houten | 07/2006 - 04/2009 | 52°01'58.8"N 5°09'57.6"E | De Bilt | 52°06'07.8"N 5°10'29.3"E | | | | | 16 | 3.0 | 4.4 | 22.1 | 7.1 | 24.0 | 14.2 | 47.8 | 837.5 | 3 | 9.1 | South | Irregular |
| | IJsselmuiden | 07/2005 - 07/2009 | 52°34'12.0"N 5°55'40.8"E | Heino | 52°25'38.2"N 6°13'18.5"E | | | | | 17 | 6.8 | 10.0 | 22.2 | 7.1 | 25.0 | 20.2 | 52.7 | 612.0 | 3 | 16.1 | South | Irregular |
| | Izmir | 1980 - 1990 | 38°24'00.0"N 27°10'12.0"E | Mulga | 37°12'00.0"N 28°21'00.0"E | Akhisar | 38°55'12.0"N 27°52'12.0"E | Dikili | 38°55'12.0"N 27°52'12.0"E | 18 | 7.0 | 1.1 | 35.0 | 14.7 | 95.0 | 16.5 | 29.0 | 4630.0 | 3 | 9.1 | South | Regular |
| | Leeuwarden | 01/2007 - 03/2009 | 53°12'21.6"N 5°48'36.0"E | Leeuwarden Airport | 53°13'09.7"N 5°45'15.8"E | | | | | 19 | 3.0 | 2.2 | 21.0 | 6.8 | 21.0 | 16.5 | 56.2 | 1287.2 | 3 | 12.1 | South - West | Regular |
| | Leiden | 03/2004 - 03/2009 | 52°09'43.2"N 4°32'24.0"E | Valkenburg | 50°51'24.7"N 5°49'59.7"E | | | | | 20 | 5.6 | 2.2 | 21.0 | 8.3 | 32.0 | 19.0 | 51.2 | 5066.6 | 3 | 15.1 | South - West | Mixed |
| | Łódź | 1992 - 1994 | 51°46'08.9"N 19°27'51.8"E | Lublinek | 51°43'23.2"N 19°23'37.1"E | | | | | 21 | 7.0 | 11.1 | 24.5 | 11.3 | 41.0 | 20.3 | 18.0 | 2330.0 | 2 | 16.0 | South | Mixed |
| | London, City centre | Summer 2000 | 51°31'08.2"N 0°07'35.6"W | London countryside | 51°32'18.0"N 0°33'10.4"W | | | | | 22 | 7.6 | 3.3 | 21.2 | 7.6 | 22.0 | 15.1 | 30.7 | 12035.0 | 2 | 11.1 | South - East | Regular |
| | London, Spitalfields | Summer 2000 | 51°31'15.9"N 0°04'14.5"W | London countryside | 51°32'18.0"N 0°33'10.4"W | | | | | 23 | 8.6 | 3.3 | 21.2 | 7.6 | 22.0 | 14.5 | 21.9 | 16057.0 | 2 | 9.1 | South | Mixed |
| | Losser | 01/2003 - 12/2008 | 52°15'18.0"N 7°00'00.0"E | Twente | 52°14'24.4"N 6°51'01.2"E | | | | | 24 | 6.8 | 1.1 | 22.7 | 12.1 | 21.0 | 19.0 | 60.9 | 3753.7 | 3 | 12.1 | South | Irregular |
| | Madrid | Summer 2008 | 40°25'46.1"N 3°42'10.9"W | Madrid Quarto Vientos | 40°22'06.5"N 3°47'06.8"W | Madrid Getafe | 40°17'51.0"N 3°43'15.2"W | Madrid Barajas | 40°29'48.6"N 3°34'25.8"W | 25 | 6.0 | 5.6 | 31.0 | 15.9 | 82.0 | 24.9 | 27.4 | 5334.0 | 1 | 8.1 | South | Mixed |
| | Moscow, City centre | Summer 2014 | 55°44'50.7"N 37°37'32.5"E | Pavlovsky | 55°46'27.2"N 38°41'55.1"E | Naro-Fominsk | 55°23'01.5"N 36°44'57.5"E | Klin | 56°18'59.2"N 36°44'26.0"E | 26 | 9.8 | 8.9 | 21.5 | 9.1 | 32.0 | 19.9 | 8.6 | 5024.3 | 1 | 14.0 | South | Mixed |
| | Padova | 2010 - 2011 | 45°24'24.4"N 11°52'49.0"E | Legnaro | 45°20'40.4"N 11°57'11.6"E | | | | | 27 | 6.0 | 7.8 | 28.3 | 10.6 | 42.0 | 19.5 | 35.9 | 2286.7 | 2 | 8.1 | South | Mixed |
| | Paris, City centre | Summer 1994 | 48°51'28.5"N 2°20'54.7"E | Paris Airport (One of the 20) | 49°01'31.1"N 2°32'30.9"E | | | | | 28 | 8.0 | 3.3 | 24.0 | 12.3 | 8.0 | 17.3 | 10.8 | 20380.0 | 1 | 10.1 | South - West | Regular |
| | Paris, Nord | Summer 1994 | 48°57'51.9"N 2°18'52.1"E | Paris Airport (One of the 20) | 49°01'31.1"N 2°32'30.9"E | | | | | 29 | 4.6 | 3.3 | 24.0 | 12.3 | 8.0 | 18.9 | 52.7 | 7010.0 | 3 | 14.1 | South - West | Regular |
| | Purmerend | 01/2008 - 03/2009 | 52°29'24.0"N 4°55'48.0"E | Berkhout | 52°38'41.2"N 5°00'18.2"E | | | | | 30 | 4.6 | 1.1 | 21.5 | 8.7 | 23.0 | 21.3 | 61.7 | 1200.0 | 3 | 18.1 | South - West | Mixed |
| | Rome, City centre | 2013 - 2017 | 41°54'29.5"N 12°29'36.7"E | Ciampino Airport | 41°47'44.6"N 12°35'17.5"E | Fiumicino Airport | 41°47'06.6"N 12°15'51.1"E | | | 31 | 6.5 | 7.8 | 28.7 | 11.2 | 72.0 | 17.5 | 35.7 | 2241.0 | 2 | 14.1 | South | Regular |
| | Rome, Roma3 | 2015 - 2016 | 41°51'16.1"N 12°28'11.7"E | Ciampino Airport | 41°47'44.6"N 12°35'17.5"E | Fiumicino Airport | 41°47'06.6"N 12°15'51.1"E | | | 32 | 4.7 | 7.8 | 28.7 | 11.2 | 72.0 | 18.3 | 54.5 | 810.0 | 2 | 20.1 | South - East | Mixed |
| | Rotterdam, City centre | Summer 2010 | 51°55'24.2"N 4°28'35.0"E | Rotterdam Airport | 51°57'23.3"N 4°26'35.3"E | | | | | 33 | 7.9 | 6.7 | 21.0 | 8.3 | 31.0 | 13.6 | 20.2 | 6116.0 | 1 | 15.1 | South | Mixed |
| | Rotterdam, East | Summer 2010 | 51°55'31.41"N 4°32'54.13"E | Rotterdam Airport | 51°57'23.3"N 4°26'35.3"E | | | | | 34 | 4.8 | 6.7 | 21.0 | 8.3 | 31.0 | 18.8 | 30.6 | 4807.0 | 3 | 15.1 | South | Mixed |
| | Rotterdam, South | Summer 2010 | 51°53'16.59"N 4°29'17.83"E | Rotterdam Airport | 51°57'23.3"N 4°26'35.3"E | | | | | 35 | 6.9 | 6.7 | 21.0 | 8.3 | 31.0 | 14.0 | 37.3 | 5227.0 | 2 | 20.1 | South | Regular |
| | Rotterdam, West | 12/2007 - 03/2009 | 51°55'01.2"N 4°25'48.0"E | Rotterdam Airport | 51°57'23.3"N 4°26'35.3"E | | | | | 36 | 9.8 | 6.7 | 21.0 | 8.3 | 31.0 | 17.4 | 35.4 | 14216.0 | 2 | 18.1 | South | Mixed |
| | The Hague | 07/2007 - 04/2009 | 52°02'24.0"N 4°14'24.0"E | Valkenburg | 50°51'24.7"N 5°49'59.7"E | | | | | 37 | 5.6 | 2.2 | 21.0 | 8.3 | 23.0 | 17.7 | 58.2 | 5161.9 | 4 | 18.1 | South | Mixed |
| | Torino, Consolata | 2009 - 2010 | 45°04'35.6"N 7°40'44.1"E | Venaria | 45°08'01.5"N 7°38'03.9"E | Caselle Airport | 45°11'38.8"N 7°38'57.5"E | Marentino | 45°03'17.4"N 7°52'45.2"E | 38 | 9.0 | 4.4 | 26.8 | 11.1 | 45.0 | 19.7 | 22.1 | 6731.3 | 2 | 8.1 | South - West | Regular |
| | Trento Molino Vittoria | Summer 2007 | 46°03'58.3"N 11°06'58.0"E | Gardolo | 46°06'22.2"N 11°06'59.2"E | Cognola | 46°04'40.8"N 11°08'38.7"E | Paganella | 46°08'59.4"N 11°02'00.2"E | 39 | 7.0 | 24.4 | 30.2 | 12.4 | 52.0 | 19.6 | 28.9 | 751.9 | 2 | 8.1 | South | Irregular |
| | Voorburg | 01/2006 - 12/2008 | 52°04'48.0"N 4°21'00.0"E | Rotterdam Airport | 51°57'23.3"N 4°26'35.3"E | | | | | 40 | 5.3 | 6.7 | 21.0 | 8.3 | 31.0 | 17.0 | 55.0 | 6332.3 | 2 | 18.1 | South - West | Mixed |
| | Wageningen | 01/2008 - 03/2009 | 51°58'12.0"N 5°40'12.0"E | Wageningen University | 51°59'07.3"N 5°39'54.2"E | | | | | 41 | 5.6 | 1.1 | 21.2 | 7.1 | 24.0 | 17.2 | 55.0 | 1154.0 | 3 | 15.1 | South | Mixed |
